# Small-Bodied Humans from Palau, Micronesia

**DOI:** 10.1371/journal.pone.0001780

**Published:** 2008-03-12

**Authors:** Lee R. Berger, Steven E. Churchill, Bonita De Klerk, Rhonda L. Quinn

**Affiliations:** 1 Institute for Human Origins and the Bernard Price Institute for Palaeontology, School of GeoSciences, University of the Witwatersrand, Johannesburg, South Africa; 2 Department of Biological Anthropology and Anatomy, Duke University, Durham, North Carolina, United States of America; 3 Department of Earth and Planetary Sciences, Rutgers University, Piscataway, New Jersey, United States of America; University of Wisconsin, United States of America

## Abstract

Newly discovered fossil assemblages of small bodied *Homo sapiens* from Palau, Micronesia possess characters thought to be taxonomically primitive for the genus *Homo*.

**Background:**

Recent surface collection and test excavation in limestone caves in the rock islands of Palau, Micronesia, has produced a sizeable sample of human skeletal remains dating roughly between 940-2890 cal ybp.

**Principle Findings:**

Preliminary analysis indicates that this material is important for two reasons. First, individuals from the older time horizons are small in body size even relative to “pygmoid” populations from Southeast Asia and Indonesia, and thus may represent a marked case of human insular dwarfism. Second, while possessing a number of derived features that align them with *Homo sapiens*, the human remains from Palau also exhibit several skeletal traits that are considered to be primitive for the genus *Homo*.

**Significance:**

These features may be previously unrecognized developmental correlates of small body size and, if so, they may have important implications for interpreting the taxonomic affinities of fossil specimens of *Homo*.

## Introduction

Living humans exhibit marked inter-populational variation in mean body size and body proportions, which reflects in part adaptive responses to variation in climatic conditions, ecological circumstances, energetics and predation risk. Within this broad pattern of human body size polymorphism are a number of cases of “pygmoid” or dwarfed populations. Pygmy populations are known from mainland tropical forests and tropical island settings in Africa and Southeast Asia [1], reflecting parallel cases of dwarfing in response to the combined factors of relative genetic isolation, a reduced resource base, hot and humid climates, hilly topography, thick undergrowth of vegetation, and (in certain island contexts) an absence of terrestrial predators [2]–[4].

Preliminary sampling of two burial caves in Palau, Micronesia has produced the remains of small-bodied recent *H. sapiens*, possibly representing a case of insular dwarfing. Individuals in this sample exhibit, in addition to small body size, reduction of the absolute size of the face, distinct supraorbital tori (in some individuals), a weakly developed mental eminence, relatively large dental dimensions, and dental dysplasias and agenesis. Some of these features may be considered primitive for the genus *Homo* (or trending towards the primitive condition), thus the human fossils from Palau may provide important insights into the relationship between small body size and the expression of morphological features generally considered to be taxonomically diagnostic in our genus. Given the scarcity of skeletal samples of small-bodied modern humans, and their importance for resolving taxonomic and phylogenetic issues in genus *Homo* paleontology, we provide here a brief description of the more salient specimens and a preliminary analysis of the material relative to small-bodied modern humans and to the holotype specimen of one small-bodied member of our genus, *H. floresiensis* (LB1).

### Geographic and archeological context

**Figure 1:**
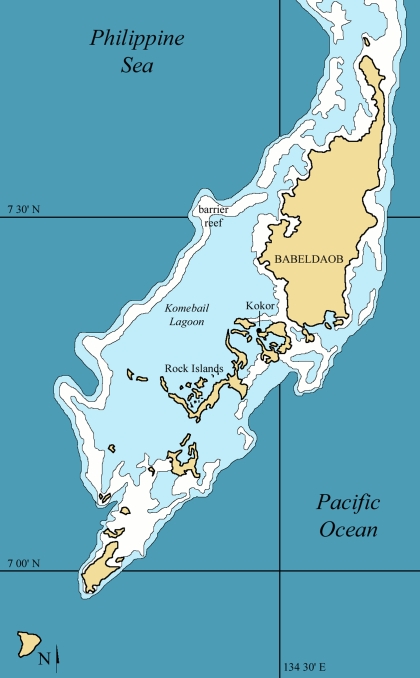
Map of Palau indicating the position of the Rock Islands to the south and southwest of the large island of Babeldaob. The caves discussed in this paper are found on western and eastern edges of the Rock Islands. Specific locations of the caves are not given for security reasons.

Palau is situated among the Western Caroline Islands on the western Pacific rim, approximately 600 km from the nearest large landmasses (Papua New Guinea to the south and the Philippines to the west). The islands that comprise the Palauan archipelago are dominated by the large, volcanic island of Babeldaob, but also include, to the south of the capital of Koror, hundreds of islets and islands of raised limestone that are colloquially known as the “rock islands” [5] (Figure 1) These rock islands contain numerous caves and rock shelters, and many of these sites contain abundant fossilized or subfossilized human remains. At least ten burial caves have been discovered in the rock islands, and excavations at one of them (Chelechol ra Orrak) has produced the skeletal remains of at least 25 individuals [6], [7] The remains discussed here were recovered from two such sites (Ucheliungs and Omedokel caves), which appear to have served exclusively as burial sites for the early inhabitants of the islands (absence of cultural remains and living debris indicates that these caves were not habitation sites).

The timing of the first human colonization of Palau is unclear. The majority of reliable radiocarbon dates from archaeological sites suggest a first occupation around 3000 cal years ago or slightly earlier, although less reliable dates have indicated a first occupation potentially as early as 4000 cal years ago [6]–[9]. Subsidence of the rock islands since mid-Holocene times, however, may have resulted in a situation in which the earliest coastal habitation sites now lie below sea level [5], [9]. Palynological evidence (in the form of a sharp increase in the amount of charcoal grains and a marked change in the mix of plant taxa represented in pollen sequences, both reflecting anthropic alteration of the native forest community) indirectly suggests a human presence possibly as early as 4500 years ago [10]; [and additional references in 5]. Regardless of the timing of the first peopling of Palau, the colonizing population most likely derived from the Philippines [see references in 11], Archeological evidence indicates that the inhabitants of Palau were in contact with their neighbors on other Western Caroline islands, but by the time of European contact Palauans were no longer engaged in voyaging to distant islands [12]. Although early Palauans were not genetically isolated, relatively low levels of gene flow from neighboring populations may have contributed to the evolution or maintenance of small body size.

The islands of Palau are devoid of indigenous terrestrial mammals and large reptiles, and prehistoric subsistence economies were based on swidden agriculture and the utilization of marine resources [5], [9], [13]. Firm archaeological evidence of fishing, primarily from near shore and lagoonal habitats, dates to only about 1700 years ago, although further sampling of early sites is likely to push this date back in time [13].

### Geochronological context and limitations of the Palauan samples

**Figure 2:**
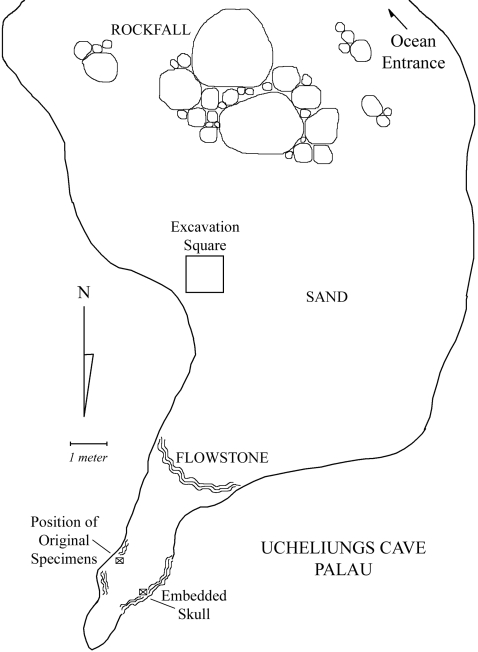
Reconstruction from a field map of the southern aspect of Ucheliungs Cave. The area where a 1×1 meter test excavation was made is indicated by the excavation square. The Position of Original Specimens marks the location where the first fossil were discovered by LRB. The Embedded Skull indicates the position of a more complete cranial specimen encased in dense flowstone.

**Figure 3:**
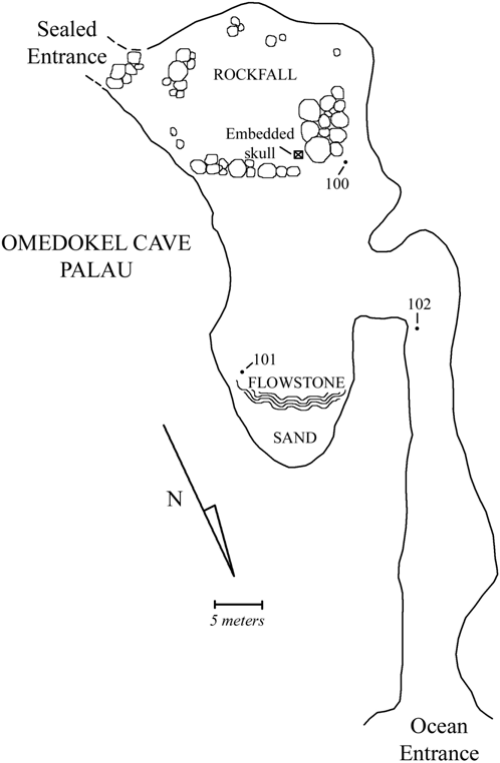
A field map of Omedokel Cave. Numbers 100–102 indicate the approximate position of samples of bone collected that yielded successful radiocarbon dates (see Supplementary Data S1). The position of the embedded skull shown in Supplementary Data S5 is indicated.

Exploration of Ucheliungs (Figure 2) and Omedokel (Figure 3) caves in 2006 revealed substantial numbers of fragmentary and complete human remains. Archaeological excavation in a 1 m×1 m×50 cm deep excavation and surface sampling in the interiors of both caves in 2006 and 2007 led to the assembling of a substantial collection of human material (Ucheliungs cave NISP (excavation and surface) >1000, and Omedokel cave NISP (surface) = 87). Based on the abundance of human bone recovered to date, future work should yield thousands of additional fragments and potentially several tens of individuals from each site.

Among this sample are a number of individuals that are small even relative to other pygmy populations (and that approximate in size *H. floresiensis* specimens and small members of the genus *Australopithecus*). 2 Sigma calibration for AMS radiocarbon dates on bone from the Ucheliungs cave surface collection and excavation range between 1420 and 2890 cal ybp, and all represent small-bodied individuals. The interior of Omedokel cave yielded small-bodied individuals dated to between 1410 and 2300 cal ybp. In the entrance to Omedokel cave, however, remains of larger individuals - in association with grave goods typical of early Palauan burials -date to between 940 and 1080 cal ybp (see Supplementary Data S1).

**Figure 4:**
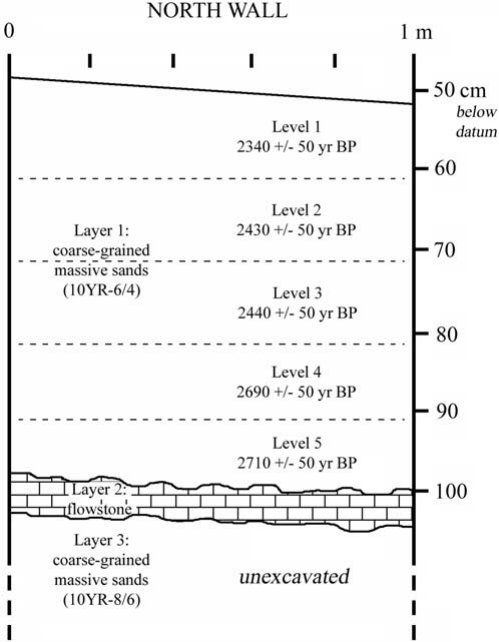
Stratagraphic column of the Ucheliungs Cave Excavation Square noted in Figure 2. Successful radiocarbon dates were obtained from human bone in levels 1–5 (see Supplementary Data S1).

The caves do not contain associated faunal remains, and cultural artefacts are rare. Given a subsidence rate of ca. 0.55 mm/year [5], the caves may have been as much as a meter and half higher during the first episodes of use of the burial caves). Human remains recovered thus far all appear to be disturbed from their primary burial context and secondarily redeposited, most likely by the action of waves that may have entered the caves during storms and/or by bioturbation. Sediments within the excavation square are course-grained sands and lack primary structures (e.g., bedding), and we found remains of modern land crabs in all excavation levels. Skeletal materials dated from all excavation levels (5 levels, 10 cm/level) maintain stratigraphic order (see Figure 4); however, due to disturbance and redeposition, we have recovered very few associated skeletal elements. Furthermore, we have discovered several relatively complete crania, but all of them are heavily embedded in calcium carbonate flowstones (and most of them remain *in situ*). These limitations make it impossible for us to make definitive statements about critical aspects of skeletal morphology in these ancient Palauans – namely brain size and body proportions (e.g., facial size relative to body size, brain size relative to body size, and relative megadontia). Because these aspects of morphology are important to the interpretation of small-bodied fossil human remains, we have tried to make what inferences we could based on preserved morphology in more fragmentary specimens and based on general size patterns in the fossil assemblage (explained below). More definitive statements must await the recovery (hopefully) of associated skeletal elements and the recovery and preparation of flowstone embedded crania.

### Taxonomic attributes of the Palauan fossils

The question may be asked if the human remains from Palau represent a case of insular dwarfing in a population of *H. sapiens*, or if – as with the inferred situation on Flores Island [14], [15]– they may represent a separate species of small-bodied humans. While the remains from Palau share with the fossils from Flores some morphological features that are primitive for the genus *Homo* (discussed below), they also possess craniofacial traits that are considered to be uniquely derived (autapomorphic) in *H. sapiens* [see *16*]. These features include a distinct maxillary canine fossa, a clearly delimited mandibular mental trigone (in most specimens), moderate bossing of the frontal and parietal squama, a lateral prominence on the temporal mastoid process, reduced temporal juxtamastoid eminences, and (based on a partial cranial vault preserving portions of the occipital and right and left parietals) an “*en maison*” cranial vault profile with greatest interparietal breadth high on the vault. Furthermore, at least one of the primitive features seen in some of the Palauan fossils – the distinct development of a supraorbital torus – is also seen in some modern human populations [17]. We feel that the most parsimonious, and most reasonable, interpretation of the human fossil assemblage from Palau is that they derive from a small-bodied population of *H. sapiens* (representing either rapid insular dwarfism or a small-bodied colonizing population), and that the primitive traits they express reflect possible pliotropic or epigenetic correlates of developmental programs for small body size. In the comparisons drawn below, we note the shared possession of these traits with the Liang Bua fossils not to imply phylogenetic affinity or taxonomic identity, but rather to caution that some of the primitive features argued to reflect an ancestor-descendant relationship between *H. erectus* and *H. floresiensis* may also be homoplastically shared with modern humans from Palau, and thus that care must be exercised in interpreting their taxonomic and phylogenetic significance.

### Body size of early Palauans

Although inclusion of the 900 ybp Omedokel burials suggests there may have been temporal variation in body size across Palauan prehistory (with later peoples being larger), we confine our discussion here to the smaller individuals recovered from surface collection in the deep interiors of the two caves and from our excavation. AMS radiocarbon dates from both caves suggest these individuals died between c2900 and 1400 years ago. The combined skeletal assemblages include specimens of subadult individuals, but all specimens analysed in this paper exhibit skeletal or dental indicators of adult developmental age (see Supplementary Data S2, S3). In the following discussion of the material, National Museum of Belau designations with -15 signify specimens from Omedokel cave, while -14 signifies specimens from Ucheliungs cave.

**Figure 5:**
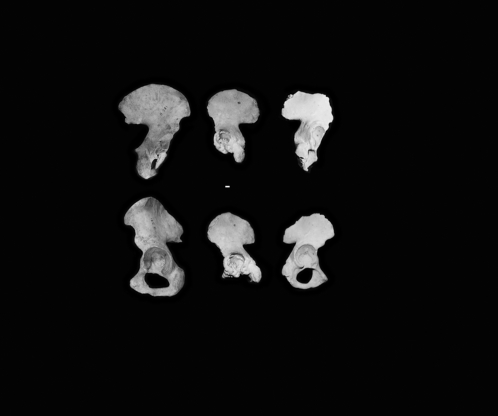
Comparison of the two innominates from Palau to that of a modern adult female of average stature (c162 cm). From left to right – modern human pelvis (top is from the right, bottom is from the left), B:OR-15:18-009 and B:OR-15:18-087. Top: posterolateral view; bottom: lateral view. Maximum iliac breadth can be calculated for both. Calcium carbonate obscures part of the acetabulum of B:OR-15:18-009. Scale bar 1 cm.

The pelvic girdle is represented by three specimens, two of which are measurable (Figure 5). B:OR-15:18-009 is a left os coxa lacking the pubis and most of the ischium. Based on the broad greater sciatic notch, this specimen likely represents a female. Calcium carbonate obscures part of the acetabulum, but the transverse diameter can be measured at 36 mm and the maximum diameter at 39.5 mm. The ilium exhibits only slight damage, and has a greatest width of 123 mm and a height of 102.3 mm, both of which approximate the smallest known *Australopithecus afarensis* (AL-288-1: Lucy) os coxa [18]. B:OR-15:18-087 is a right os coxa lacking most of the ischium but retaining much of the pubic bone. The narrow sciatic notch and details of pubic morphology suggest that B:OR-15:18-087 represents a male. B:OR-15:18-009 is very close in size to the Flores LB1 os coxa, while B:OR-15:18-087 is slightly larger, and has a maximum acetabular diameter of 46.1 mm. None of the three specimens exhibit the marked lateral flare observed in the *H. floresiensis* ilium, but rather compare favourably with the morphology of larger modern *H. sapiens*. In maximum diameter of the acetabulum the early Palauan mean falls very close to the mean value from 17 Andamanese pygmies (Table 1), and the sample means from the two groups are not significantly different.

**Table 1:** Craniofacial and postcranial dimensions (mm) in Palauans, small-bodied Andamanese and Africans (San)^a^, and Flores LB1^b^

**Facial dimensions**	
	**Orbital Breadth** c
B:OR-14: 8-001	31.0
B:OR-15:18-005	36.8
B:OR-15:18-080	37.0
B:OR-15:18-081	32.5
Palauan average (mean, SD, n)	34.3±3.0 (4)
San, pooled sexes (mean, SD, n)	36.8±3.0 (13)
Flores (LB1)	32
	**Interorbital Breadth** d
B:OR-14: 8-001	26.9
B:OR-15:18-005	23.8
B:OR-15:18-080	29.0
B:OR-15:18-081	25.9
Palauan average (mean, SD, n)	26.4±2.2 (4)
San, pooled sexes (mean, SD, n)	24.9±2.9 (12)
	**Nasal Breadth** e
B:OR-14:8-1016	20.2
B:OR-15:18-051	22.0
B:OR-15:18-086	21.4
B:OR-15:18-084	25.6
Palauan average (mean, SD, n)	22.3±2.3 (4)
San, pooled sexes (mean, SD, n)	26.3±2.2 (12)
Flores (LB1)	21
	**Mandibular Symphyseal Height** f
B:OR-14:8-122	26.6
B:OR-15:18-001	30.0
B:OR-15:18-008	27.7
B:OR-15:18-083	35.6
Palauan average (mean, SD, n)	30.0±4.0 (4)
San, pooled sexes (mean, SD, n)	30.3±4.4 (5)
Flores (LB1)	28
	**Mandibular Mental Foramen Height** g
B:OR-14:8-108	22.0
B:OR-14:8-122	27.1
B:OR-14:8-771	23.0
B:OR-15:18-008	29.5
B:OR-15:18-083	35.4
	
Palauan average (mean, SD, n)	27.4±5.4 (5)
San, pooled sexes (mean, SD, n)	27.6±3.9 (5)
	**Mandibular Molar Height** h
B:OR-14:8-122	23.0
B:OR-14:8-771	22.6
B:OR-15:18-006	21.9
B:OR-15:18-008	25.1
B:OR-15:18-083	32.0
Palauan average (mean, SD, n)	24.9±4.1 (5)
San, pooled sexes (mean, SD, n)	24.9±3.5 (5)
Flores (LB1)	20.5
Postcranial dimensions	
	**Humeral Distal Articular Breadth** i
B:OR-14:8-991	34.9
B:OR-15:18-014	32.9
B:OR-15:18-015	34.9
B:OR-15:18-024	44.1
B:OR-15:18-054	41.2
B:OR-15:18-088	42.4
Palauan average (mean, SD, n)	38.4±4.7 (6)
San, pooled sexes (mean, SD, n)	42.1±2.0 (9)
	**Acetabular Maximum Diameter** j
B:OR-15:18-009	39.5
B:OR-15:18-087	46.1
Palauan average (mean, SD, n)	42.8±4.7 (2)
Andamanese, pooled sexes (mean, SD, n)	42.6±2.6 (17)
San, pooled sexes (mean, SD, n)	47.7±3.4 (11)
Flores (LB1)	36
	**Femoral Head AP Diameter** k
B:OR-15:18-013	36.1
B:OR-15:18-098	38.8
Palauan average (mean, SD, n)	37.5±1.9 (2)
Andamanese, pooled sexes (mean, SD, n)	37.3±2.4 (38)
San, pooled sexes (mean, SD, n)	42.3±3.3 (10)
Flores (LB1)	31.5
	**Tibial Proximal AP Diameter** l
B:OR-14:8-003	34.4
B:OR-15:18-040	32.5
Palauan average (mean, SD, n)	33.5±1.3
San, pooled sexes (mean, SD, n)	43.9±3.1 (10)
	**Tibial Proximal ML Diameter** m
B:OR-14:8-003	63.1
B:OR-15:18-040	53.1
Palauan average (mean, SD, n)	58.1±7.1 (2)
Andamanese, pooled sexes (mean, SD, n)	57.8±7.8 (30)
San, pooled sexes (mean, SD, n)	67.7±5.6 (10)
Flores (LB1)	51.5
	**Talar Length** n
B:OR-14:8-109	51.3
B:OR-15:18-010	43.7
B:OR-15:18-011	52.3
B:OR-15:18-038	43.8
B:OR-15:18-039	46.4
Palauan average (mean, SD, n)	47.5±4.1 (5)
San, pooled sexes (mean, SD, n)	53.2±5.0 (9)

a. Andaman and Nicobar Islanders from the collections of Natural History Museum (London) and Cambridge University (Duckworth Collection), and Kalahari San (Bushmen) from the collections of the University of the Witwatersrand were used as small-bodied comparative groups. All comparative data collected by BDK.

b. Data for LB 1 from P. Brown *et al.*, *Nature* **431**, 1055–1061 (2004) and accompanying supplemental data.

c. Breadth of the orbit from ectoconchion to dacryon (the frontal process of the zygomatic was missing in all cases, but ectoconchion could still be determined as it falls at the frontomaxillary juncture).

d. Breadth across the nasal space from dacryon to dacryon.

e. Maximum distance between the anterior edges of the nasal aperture (in most cases this dimension was estimated by doubling the distance from the midline of the nasal aperture [as determined by the anterior nasal spine and midmaxillary suture] to the edge of the nasal aperture on one side).

f. M-69 [**34**].

g. M-69(1) [**34**].

h. M-69(2) [**34**].

i. M-12a [**34**].

j. from the acetabular margin immediately adjacent to the middle of the anterior inferior iliac spine to the most distant point on the inferior margin.

k. M-19 [**34**].

l. M-8a [**34**].

m. M-9a [**34**].

n. M-1 [**34**]

Body mass was estimated from maximum acetabular diameter following the method described in [19], and using a mass estimation regression appropriate to small-bodied humans [that of 20]; [ see 21]. This method produced mass estimates of 28.7 kg for the female specimen and 43.2 kg for the male. The same regression applied to small samples of Onge from the Andaman and Nicobar Islands (collections of the Natural History Museum, London and the Cambridge University Duckworth collection) produced female and male mean estimates of 40.1 kg (±3.7 kg, n = 12) and 47.4 kg (±5.2 kg, n = 12), respectively. Tests of a single variate against a sample mean (a modified instance of the t-test: [22]) reveal that the female mass estimate is significantly different (that is, p<0.05 of being drawn from a population with a body mass mean and variance equal to that of Onge sample) than the mean value for Onge females, whereas the male mass estimate is not significantly different from the Onge male average.

Two proximal femoral fragments were sufficiently preserved to allow measurement of the femoral head. B:OR-15:18-013 has a superoinferior femoral head diameter of 35.2 mm, and a long neck relative to femoral head size (biomechanical neck length 36.1 mm). Following [20], the body mass predicted from this femoral head is 38.9 kg. A second partial femoral head, B:OR-15:18-098, has an anteroposterior head diameter of 38.8 mm. Femoral head anteroposterior diameters tend to be slightly larger than superoinferior diameters, and would thus be expected to produce slightly larger mass estimates. Using the anteroposterior diameter we estimate body mass of this individual at 47 kg. The sex of the individuals represented by these specimens is unknown, and therefore we compare these mass estimates to the mean mass estimate derived from a pooled sex sample of Onge femora (12 females, 12 males and 14 specimens of indeterminate sex). The mean estimate derived from the two Palauan femora (43.0±5.7 kg) does not differ significantly from the pooled sex Onge mean (43.7±5.3 kg, n = 38).

Two measurable proximal tibial specimens were recovered. B:OR-14:8-003 has a bicondylar breadth of 63.1 mm, which falls above (and is not significantly different from) the mean of a pooled sex sample of Onge (57.8±7.8 mm, n = 29). The other specimen, B:OR-15:18-040, has a bicondylar breadth of 53 mm, which falls below the Onge mean but again is not significantly different. This specimen reflects an individual of similar size to Liang Bua 1 (which has a bicondylar breadth = 51.5 mm).

**Figure 6:**
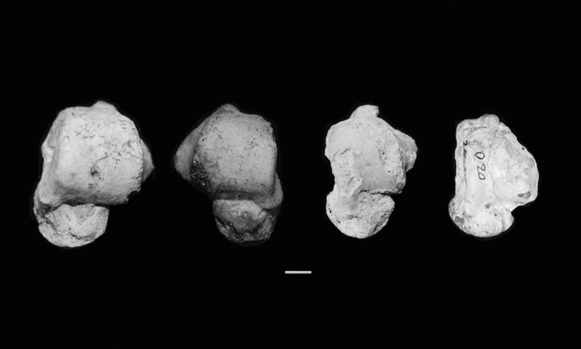
Three tali from Palau (left) illustrating variation in size and shape. The “Little Foot” talus (right) - from a very small bodied early hominin from Sterkfontein, South Africa [32]- is included for comparison. From left to right B:OR-15:18-010 the largest talus collected; B:OR-15:18-011 illustrating a medium sized talus from Palau; B:OR-15:18-039 representing a smaller bodied individual; Little Foot. Of nine tali recovered, at least two individuals have some joint dimensions smaller than those of Little Foot. Scale bar 1 cm.

More than 61 measurable postcranial elements recovered from the two caves also indicate body sizes at the lower extreme of recent human variation and in some cases the range of small-bodied australopithecines. These include tali that approach closely the size of the talus of the small bodied australopithecine “Little Foot” from South Africa (Figure 6), supporting the hypothesis that small body size was the norm in the earlier populations preserved in the cave. We are careful to point out, however, that body size estimation techniques generally have large associated errors of estimation, especially at the extreme ends of the size ranges of the samples that were used to develop the techniques. Still, the rough estimates above provide a tentative indication of the small size of the early Palauans.

### Cranio-facial size and morphology of early Palauans

The adult cranial sample collected to date from Ucheliungs and Omedokel caves is represented by more than 30 cranial fragments. More complete crania are present, but as mentioned are embedded in dense flowstone or are still *in situ* and may take many years to prepare. As with the postcranial analysis, specimens from the older stratigraphic layers of the excavation and deeper cave interiors are the focus of this discussion.

**Figure 7:**
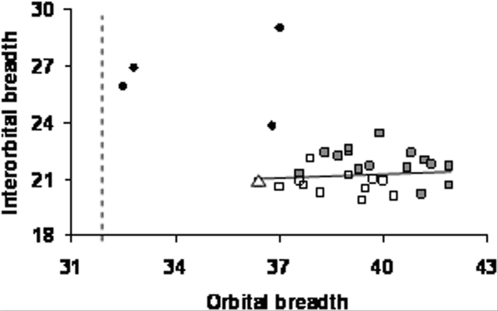
Interorbital breadth (mm) versus orbital breadth (mm) in Australasians. Filled circles: Palauan specimens; filled squares: sample means for recent human males; open squares: sample means for recent human females; open triangle; sample mean for Andamanese pygmy females; dotted line: orbital breadth value for LB1 (from supplemental data in [1]). Least squares regression line (y = 0.0624x+18.913, r = 0.1044) based on recent human sample means only. Recent human data representing Australian, Melanesian, Polynesian, western Pacific and Far Eastern populations from WW Howells [33].

Orbital dimensions are small even relative to female pygmies from the Andaman Islands (Figure 7), and average orbital and nasal breadth values in the Palauans fall below mean values for a comparative sample of small-bodied modern humans (San bushmen from the Kalahari) and fall close to reported values for LB1 (see Table 1). A similar pattern is seen in mandibular dimensions (Table 2 & 3). While facial dimensions in the Palauan sample are absolutely smaller than those of the San (at the time of preparation of the manuscript, comparative data collection was still underway, thus comparison is made here only to the African San sample), their faces were larger relative to body size. Associated craniofacial and postcranial elements are lacking in the Palauan sample, making it difficult to evaluate facial-to-body size proportions. As a heuristic measure, we created facial size ratios by pairing the smallest value for a given facial variable with the smallest value for a given postcranial variable, and by pairing the largest facial dimension with the largest postcranial dimension for each variable (see Supplementary Data S4). We will no doubt be criticized for constructing what might be considered unreasonable chimeras from unassociated cranial and postcranial remains, but we reiterate that, given the lack of associated material in the assemblage, we engage in this exercise simply as a means of exploring facial size in the assemblage *vis a vis* the size of postcranial remains in the assemblage, following the logic developed by [23] for unassociated upper and lower limb remains of *Australopithecus africanus.* In every case the resulting facial size/body size ratios are larger than the mean ratio of the same variables in the San

**Table 2:** Mandibular dimensions in the Palauan sample, LB1 and recent modern humans (recent *H. sapiens* data from supplementary material in [14]).

	Symphyseal height (mm)	Symphyseal thickness (mm)	Corpus height at M_1_/M_2_ (mm)	Corpus thickness at M_1_/M_2_ (mm)
Palauan sample	30.0	14.6	25.6	14.6
	4.0	1.0	4.0	1.6
	(4)	(4)	(6)	(8)
LB1	28	15	20.5	15.5
Recent *H. sapiens*	33.8	15.1	29.3	14.3
	3.7	1.7	3.2	1.7
	(1050)	(530)	(478)	(508)

Mean, SD,(n)

**Table 3:** Buccolingual crown breadth (mm) in the Palauan sample, LB1 and recent modern humans (recent *H. sapiens* data from supplementary material in [14]).

	I1	I2	C	P3	P4	M1	M2	M3
**Maxillary**								
Palau	7.1	6.8	9.7	9.9	9.9	12.3	12.3	11.7
	0.2	0.9	4.0	0.5	0.4	1.1	1.0	
	(5)	(10)	(9)	(11)	(6)	(7)	(6)	(1)
LB1	-	-	8.7	10.3	9.3	11.9	11.4	-
*H. sapiens* male	-	-	8.7	9.7	9.6	11.8	12.1	-
			0.7	0.8	0.8	1.0	1.2	
			(696)	(474)	(492)	(505)	(729)	
*H. sapiens* female	-	-	8.2	9.4	9.2	11.5	11.6	-
			0.7	0.8	0.7	0.9	1.1	
			(501)	(244)	(293)	(295)	(538)	
**Mandibular**								
Palau	6.4	6.1	7.8	8.6	8.8	11.4	11.0	11.0
	0.3	0.6	0.3	0.5	0.8	0.5	0.5	
	(6)	(6)	(5)	(10)	(4)	(9)	(8)	(1)
LB1	5.7	6.3	7.8	8.4	-	11.1	10.7	10.2
*H. sapiens* male	5.9	6.3	8.1	8.3	-	10.9	10.7	10.7
	0.5	0.5	0.6	0.7		0.8	0.9	0.9
	(348)	(402)	(699)	(465)		(434)	(668)	(372)
*H. sapiens* female	5.7	6.1	7.4	8.0	-	10.7	10.4	10.2
	0.5	0.5	0.6	0.7		0.9	0.9	0.9
	(188)	(226)	(500)	(239)		(225)	(463)	(200)

Mean, SD, (n).

One criticism of the initial interpretation of the Flores fossils is the argument that *H. floresiensis* might actually be a pygmy *H. sapiens* [1], [4]. This criticism has been countered by the claim that known pygmies possess absolute craniofacial dimensions within the range of larger-bodied populations, while *H. floresiensis* shows reduction of the craniofacial skeleton that is proportional to their reduction in body size [14]. When the size of facial elements is considered relative to the size of postcranial elements, we observe in the Palauan sample a pattern of reduction in the craniofacial skeleton similar to that seen in LB1. It is also interesting to note that in every case for which a craniofacial size to postcranial size comparison could be made with LB1, the Flores specimen also produced a ratio greater than the mean for the San, and in most cases greater than the ratio observed in the Palauans. Thus it seems that the Palauan sample, while having facial dimensions that are absolutely smaller than our small-bodied comparative sample, has faces that are large relative to body size. However, the same must be said of LB1.

**Figure 8:**
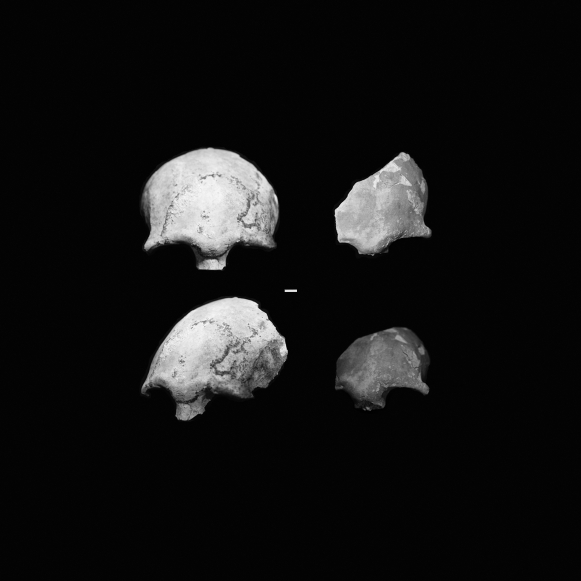
Frontal and lateral-oblique views of B:OR-15:18-001 (Right, top and bottom) and B:OR-14:8-005 (Left, top and bottom), illustrating two adult specimens with inflated glabellae and thickened lateral superciliary arches. In B:OR-15:18-005 the inflated glabellar region gives rise to curved and laterally extending projections that are continuous with thickened lateral superciliary arches, creating a moderately projecting, double curved supraorbital torus. Specimen B:OR-14:8-001 has an inflated glabellar area and thickened superciliary arches continuing laterally. However, in this specimen the inflated glabellar region is not continuous with the superciliary arches and does not give the appearance of continuous brow ridges.

While maxillary, orbital and supraorbital morphology varies within the assemblage, several unifying morphological features are notable and serve to unify the sample. All of the frontals (n = 5) have well-developed and projecting glabellar regions. The early Palauan maxillae and frontals exhibit narrow orbital and nasal breadths, combined with large interorbial breadths, when compared to small bodied populations of modern humans (see Table 1). Most specimens exhibit some degree of robusticity of either the lateral or medial portions of the supraorbital region. The frontal B:OR-14:8-001 (Figure 8, right) exhibits the swelling of the glabellar area characteristic of the sample, along with a degree of thickening of the lateral trigone. B:OR-15:18-005 (Figure 8, left).exhibits a relatively superoinferiorly and anteroposteriorly pronounced supraorbital torus that arches over the orbits and has strongly projecting lateral trigones. It is not always possible to be sure of the developmental status of isolated frontal fragments, and it is possible that one or both of these individuals represent juveniles. If that is the case, however, we would expect the adult morphology, if anything, to exhibit even more strongly projecting brow ridges.

**Figure 9:**
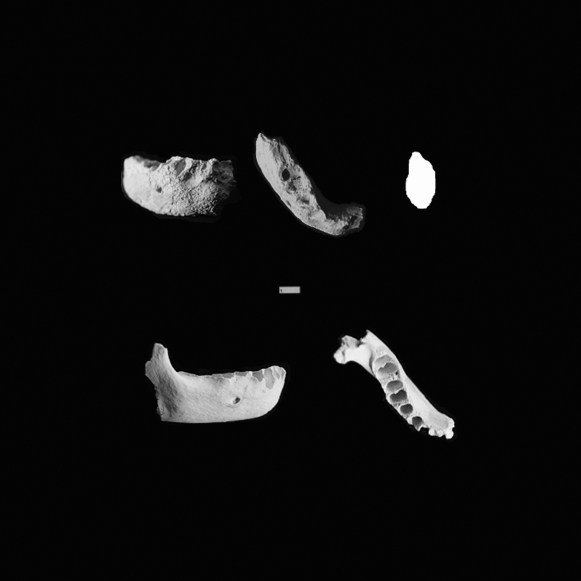
Occlusal and lateral views of B:OR-14:8-122 (top left and top middle) and B:OR-14:8-771 (bottom left and right), illustrating two adult specimens with reduced chins in the Palauan sample. B:OR-14:8-122 lacks a vertical keel as well as the distended inferior margin, and consequently lacks the T-shaped mental trigone and associated mental fossae characteristic of modern human chins. The cross section (top right) - approximately through the symphysis - clearly shows the reduction of the chin in this individual. Although part of the symphyseal region of B:OR-14:8-771 is missing, the lack of swelling of the inferior margin at the level of the canine suggests a highly reduced mental eminence. B:OR-14:8-771 also exhibits a congenitally absent third molar, a feature also common in the Palauan sample.

The Palauan mandibles are small yet robust (Table 2), and exhibit vertically-set mandibular symphyses. Post-depositional damage to most of the mandibles often obscures the morphology of this region and makes direct comparisons with Flores difficult. Nevertheless, it is clear that most of the mandibles exhibit a distinct mental trigone, but generally with weakly developed or absent mental fossae and weakly developed, non-projecting mental tubercles. The symphyseal region tends to be vertically oriented, and the mean symphyseal angle [24] (92.5°±3.1°, n = 4) is closer to that of archaic humans (90°±5.1°, n = 10) than that of recent humans (range of means: 98.5°–106.1°: [24]). The mean symphyseal angle of the Palauan sample is significantly different at p<0.001 from the mean values of each of the recent modern human samples – Bushman, Zulu and Tolai – reported in [24]. While most specimens exhibit a clearly delineated mental trigone, the weak development of the mental tubercles and the vertical orientation of the symphyses combine to produce a non-projecting mental eminence. One specimen, B:OR-14:8-122, lacks a mental trigone and other features of the human chin (central keel, mental fossae and incurvatio mandibularis: [25]), although it has a small, superiorly displaced mental tubercle (Figure 9). The two mandibular specimens from Flores (LB1 and LB6/1) have been described as lacking chins [14], [15], and LB 6/1 has been described as lacking a mental protuberance, mental tubercles and incurvatio mandibularis [15]. Others who have seen the material [supporting information in 4] have scored LB1 as having a slight incurvatio mandibularis. We have not personally examined the fossil material from Flores, but we note that the morphology of LB1 – as scored by [4] – would be consistent with that seen in most of the Palauan mandibles.

**Figure 10:**
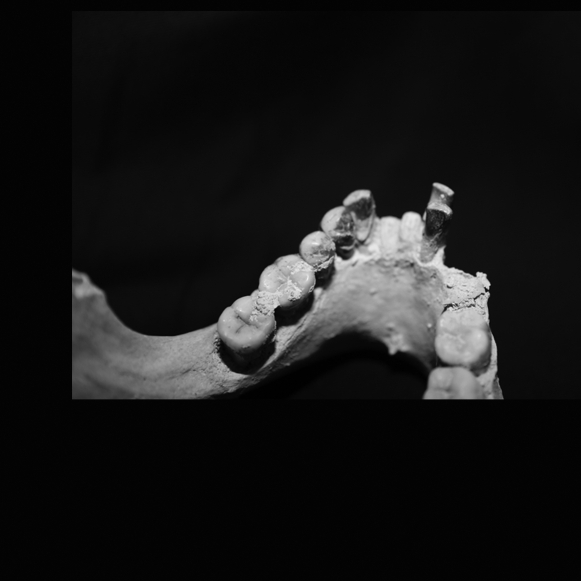
Palauan mandible B:OR-15:18-083 illustrating a number of characters common in the sample, including dental crowding of the anterior teeth, incisiform canines, caniniform premolars, large tooth size, and absence of a third molar.

The teeth from both Ucheliungs cave and Omedokel cave are large in comparison to modern humans and those of LB1 (Table 3). Estimated megadontia quotients [20] range from 1.09 to 1.31 (using estimated body mass, as determined above, of the largest and smallest individual in the sample and the largest and smallest teeth, respectively. The use of less conservative body mass estimates such as those used to estimate a 16–28.7 kg mass for *H. floresiensis* [14] yield megadontial quotients for the Palauan sample approaching those found for Flores: MQ = c1.5–1.7). The large teeth may be due to a time lag in the reduction of tooth size relative to body size [26]. Although in the mandibular sample there is typically a large space distal to the last erupted molar, third molar agenesis is frequent in both the mandible and maxilla (57% absence in mandibles [n = 7]; 66% absence in maxillae [n = 3]). Dental agensis was established by radiography, as was the adult status of the mandibular and maxillary specimens. When present, the fully erupted third molar is always malrotated. Crowding of teeth and resultant malocclusion is common. Other dental differences, most commonly involving rotation of individual teeth, have been noted in molars, premolars, canines and incisors in the Palau sample. At least two individuals show instances of caniniform P3's and incisiform canines (Figure 10). In our sample of 12 P_3_s, one specimen exhibits occlusal morphology similar to that of Flores LB1 [14] in having a relatively great occlusal surface area (molariform) and a prominent protoconid and broad talonid.

### Brain size

The only crania complete enough to allow determination of endocranial volume are heavily encased in flowstone, which has deterred our best efforts to estimate brain size in the Palauan sample (see Supplementary Data S5). Nevertheless, it is clear from these specimens that the brain size is small, possibly at the very low end or below that typically observed in modern small-bodied humans. Other recovered cranial remains are fragmentary, and accurate endocranial volumes have not yet been established for this sample. We have however, attempted (with varying degrees of success) to estimate cranial capacity through correlating three facial measurements with endocranial capacities in a large sample of modern humans (see Supplementary Data S6, S7, S8). Our results show clearly that the average endocranial volume of the Palauan sample recovered to date will almost certainly fall below the low end of the range (1000 cc) of our sample of 147 modern humans (which includes small bodied modern humans). Based upon these results and the size and morphology of other recovered neurocranial elements, brain sizes – while not ape-like as seen in LB1 – are likely at or below the low end of small bodied modern human variation, but within that of *H. erectus*.

## Discussion

We have described here a new sample of small-bodied *H. sapiens* from Micronesia. The Palauan sample also has individuals that exhibit a number of characters normally associated with more primitive species of the genus *Homo*. The modern human skeletal remains from Palau, in conjunction with pygmoid populations across Australasia, exemplify the regularity with which small body size – physiological dwarfing - emerges in island contexts (and at times in mainland populations), and the Palauen sample contributes to our understanding of human size and morphological variation in island populations. Apprehending the full nature of regional variation in Austromelanesian and Pacific Island populations is essential to interpreting the taxonomic status and phylogenetic history of *H. floresiensis*. A number of the individual traits observed in the Palauan sample are seen also in specimens from Flores (although the form of these traits may differ in the Palauan sample), some of which have been argued to support the unique taxonomic status of *H. floresiensis*: small body size, reduction of the absolute size of the face, pronounced supraorbital tori, non-projecting chins, relative megadontia, expansion of the occlusal surface of the premolars, rotation of teeth within the maxilla and mandible, and dental agenesis. These last two features are not argued to be taxonomic markers, but their occurance in specimens from both Palau and Flores is notable, as they may be parallel results of founder effects, genetic isolation and a high inbreeding coefficient, or may simply be a factor of evolutionarily rapid reduction in body and craniofacial size [26]. While we have not seen in the Palauan sample the extremely small brain size documented for one individual from Flores, current indications place the cranial capacity of the Palauan sample at the lower end (and possibly below) the range of variation presently considered “normal” in modern *H. sapiens*, and within the range of *H. erectus*

In the sample recovered to date, we have not observed all of the features used to originally define *H. floresiensis* (such as ape-like endocranial volumes and pronounced canine juga), nor would we expect to - particularly if these features were the manifestation of a genetic anomaly, such as microcephalic osteodysplastic primordial dwarfism, manifest in the type specimen LB1 [see 4], [27]–[29 vs. 1,30,31]. We have not yet recovered adequate material to compare features of the cranial base considered taxonomically important in Flores, nor do the two Palauan *ossa coxae* exhibit the extreme lateral flaring of the ilium as observed in LB1 [14].

Our findings do suggest, however, that a number of the morphological features considered either primitive for the genus *Homo* (e.g., small brain size, enlarged supraorbital tori, and absence of chins) or unique to *H. floresiensis* within the genus *Homo* (e.g., relative megadontia) may emerge as developmental correlates of small body size in pygmoid populations. This finding would be consistent with the argument that Flores LB1 may represent a congenitally abnormal individual drawn from a small-bodied island population of *H. sapiens*. These results also suggest that the simple presence of additional small-bodied specimens with reduced chins (that cannot be shown to share all of the traits considered taxonomically significant in the Holotype Flores LB1) is insufficient to confirm the taxonomic validity of *H. floresiensis*.

Based on the evidence from Palau, we hypothesize that reduction in the size of the face and chin, large dental size and other features noted here may in some cases be correlates of extreme body size reduction in *H. sapiens*. These features when seen in Flores may be best explained as correlates of small body size in an island adaptation, regardless of taxonomic affinity. Under any circumstances the Palauan sample supports at least the possibility that the Flores hominins are simply an island adapted population of *H. sapiens*, perhaps with some individuals expressing congenital abnormalities.

## Supporting Information

Supplementary Data S1 (0.03 MB DOC)

Supplementary Data S2 A summary table of the NISP (number of individual specimens) collected from Ucheliung Cave as well as a complete list of all specimens found in Ucheliung cave indicating whether the specimens were measurable as well as those specimens used for AMS and DNA analysis.
(2.27 MB DOC)

Supplementary Data S3 A summary table of the NISP (number of individual specimens) collected from Omedokel Cave as well as a complete list of all specimens found in Omedokel cave indicating whether the specimens were measurable as well as those specimens used for AMS and DNA analysis.
(0.27 MB DOC

Supplementary Data S4 (0.19 MB DOC)

Supplementary Data S5 (3.11 MB DOC)

Supplementary Data S6 (0.05 MB DOC)

Supplementary Data S7 (0.06 MB DOC)
