# Correction: Small-Bodied Humans from Palau, Micronesia

**DOI:** 10.1371/annotation/0b1a375a-ea43-4586-824f-05bc5b359d63

**Published:** 2008-03-19

**Authors:** Lee R. Berger, Steven E. Churchill, Bonita De Klerk, Rhonda L. Quinn

There is an error in the first affiliation. It should appear:

Institute for Human Evolution and the Bernard Price Institute for Palaeontology, School of GeoSciences, University of the Witwatersrand, Johannesburg, South Africa.

