# Supplementary material for: Small-Bodied Humans from Palau, Micronesia
Source: PLoS One. 2008 Mar 12;3(3):e1780. doi: 10.1371/journal.pone.0001780 (PMC2268239; doi:10.1371/journal.pone.0001780)
Supplement: Supplementary Data S1 — (0.03 MB DOC) [file pone.0001780.s001.doc]

**Supplementary Data 1**

Radiocarbon data on bone. Dating conducted by Beta Analytic, Miami Florida.

**Sample Data Measured 13C/12C Conventional**

**Radiocarbon Age Ratio Radiocarbon Age**

________________________________________________________________

B:OR-14:8-1200 2550 +/- 50 BP -15.1 o/oo 2710 +/- 50 BP

15N/14N = +11.4 o/oo

ANALYSIS : AMS-Standard delivery

MATERIAL/PRETREATMENT : (bone collagen): collagen extraction: with alkali

2 SIGMA CALIBRATION : Cal BC 940 to 800 (Cal BP 2890 to 2750)

B:OR-14:8-1201 2530 +/- 50 BP -15.3 o/oo 2690 +/- 50 BP

15N/14N = +11.4 o/oo

ANALYSIS : AMS-Standard delivery

MATERIAL/PRETREATMENT : (bone collagen): collagen extraction: with alkali

2 SIGMA CALIBRATION : Cal BC 920 to 790 (Cal BP 2870 to 2740)

B:OR-14:8-1202 2280 +/- 50 BP -15.0 o/oo 2440 +/- 50 BP

15N/14N = +11.5 o/oo

ANALYSIS : AMS-Standard delivery

MATERIAL/PRETREATMENT : (bone collagen): collagen extraction: with alkali

2 SIGMA CALIBRATION : Cal BC 780 to 400 (Cal BP 2730 to 2350)

B:OR-14:8-1203 2260 +/- 50 BP -14.7 o/oo 2430 +/- 50 BP

15N/14N = +11.8 o/oo

ANALYSIS : AMS-Standard delivery

MATERIAL/PRETREATMENT : (bone collagen): collagen extraction: with alkali

2 SIGMA CALIBRATION : Cal BC 780 to 390 (Cal BP 2730 to 2340)

B:OR-14:8-1204 2190 +/- 50 BP -15.9 o/oo 2340 +/- 50 BP

15N/14N = +13.0 o/oo

ANALYSIS : AMS-Standard delivery

MATERIAL/PRETREATMENT : (bone collagen): collagen extraction: with alkali

2 SIGMA CALIBRATION : Cal BC 520 to 360 (Cal BP 2460 to 2320)

B:OR-14:8-1205 2400 +/- 40 BP -14.4 o/oo 2570 +/- 40 BP

15N/14N = +12.6 o/oo

ANALYSIS : AMS-Standard delivery

MATERIAL/PRETREATMENT : (bone collagen): collagen extraction: with alkali

2 SIGMA CALIBRATION : Cal BC 810 to 750 (Cal BP 2760 to 2700) AND Cal BC 690 to 660 (Cal BP 2640 to 2610)

Cal BC 640 to590 (Cal BP 2590 to 2540)

B:OR-14:8-1206 1520 +/- 40 BP -17.0 o/oo 1650 +/- 40 BP

15N/14N = +13.0 o/oo

ANALYSIS : AMS-Standard delivery

MATERIAL/PRETREATMENT : (bone collagen): collagen extraction: with alkali

2 SIGMA CALIBRATION : Cal AD 260 to 280 (Cal BP 1680 to 1670) AND Cal AD 330to 450 (Cal BP 1620 to 1500)

Cal AD 450 to 460 (Cal BP 1500 to 1490) AND Cal AD 480 to 530 (Cal BP 1470 to 1420)

B:OR-14:8-1207 1570 +/- 40 BP -14.0 o/oo 1750 +/- 40 BP

15N/14N = +13.0 o/oo

ANALYSIS : AMS-Standard delivery

MATERIAL/PRETREATMENT : (bone collagen): collagen extraction: with alkali

2 SIGMA CALIBRATION : Cal AD 210 to 390 (Cal BP 1740 to 1560)

B:OR-15:18-100 1500 +/- 40 BP -17.0 o/oo 1630 +/- 40 BP

15N/14N = +10.3 o/oo

ANALYSIS : AMS-Standard delivery

MATERIAL/PRETREATMENT : (bone collagen): collagen extraction: with alkali

2 SIGMA CALIBRATION : Cal AD 340 to 540 (Cal BP 1610 to 1410)

B:OR-15:18-101 2000 +/- 40 BP -16.5 o/oo 2140 +/- 40 BP

15N/14N = +9.2 o/oo

ANALYSIS : AMS-Standard delivery

MATERIAL/PRETREATMENT : (bone collagen): collagen extraction: with alkali

2 SIGMA CALIBRATION : Cal BC 360 to 290 (Cal BP 2300 to 2240) AND Cal BC 240 to 50 (Cal BP 2180 to 2000)

B:OR-15:18-102 940 +/- 40 BP -14.6 o/oo 1110 +/- 40 BP

15N/14N = +13.0 o/oo

ANALYSIS : AMS-Standard delivery

MATERIAL/PRETREATMENT : (bone collagen): collagen extraction: with alkali

2 SIGMA CALIBRATION : Cal AD 870 to 1010 (Cal BP 1080 to 940)
