# Supplementary material for: Small-Bodied Humans from Palau, Micronesia
Source: PLoS One. 2008 Mar 12;3(3):e1780. doi: 10.1371/journal.pone.0001780 (PMC2268239; doi:10.1371/journal.pone.0001780)
Supplement: Supplementary Data S2 — A summary table of the NISP (number of individual specimens) collected from Ucheliung Cave as well as a complete list of all specimens found in Ucheliung cave indicating whether the specimens were measurable as well as those specimens used for AMS and DNA analysis. (2.27 MB DOC) [file pone.0001780.s002.doc]

| **Element(s)** | **Surface** | **Cave Extension** | **Excavation Level 1** | **Excavation Level 2** | **Excavation Level 3** | **Excavation Level 4** | **Excavation Level 5** | **Unknown Provenience** |
| --- | --- | --- | --- | --- | --- | --- | --- | --- |
| **Cranium** | 6 | 45 | 34 | 19 | 8 | 12 | 4 | 0 |
| **Mandible** | 2 | 1 | 2 | 0 | 0 | 1 | 0 | 0 |
| **Vertebra** | 2 | 1 | 13 | 9 | 11 | 6 | 0 | 0 |
| **Rib** | 4 | 1 | 33 | 11 | 28 | 33 | 4 | 0 |
| **Sternum** | 0 | 0 | 0 | 0 | 1 | 0 | 0 | 0 |
| **Scapula** | 2 | 0 | 2 | 0 | 0 | 0 | 0 | 0 |
| **Clavicle** | 0 | 0 | 0 | 1 | 1 | 0 | 0 | 0 |
| **Humerus** | 0 | 1 | 0 | 0 | 2 | 2 | 1 | 0 |
| **Radius** | 0 | 1 | 1 | 1 | 1 | 0 | 0 | 0 |
| **Ulna** | 0 | 0 | 0 | 0 | 1 | 0 | 1 | 0 |
| **Carpals** | 0 | 0 | 7 | 3 | 9 | 3 | 2 | 0 |
| **Hand phalanges** | 0 | 0 | 8 | 18 | 27 | 6 | 7 | 0 |
| **Os coxa** | 0 | 1 | 0 | 0 | 3 | 0 | 0 | 0 |
| **Sacrum/coccyx** | 0 | 0 | 1 | 0 | 0 | 0 | 0 | 0 |
| **Femur** | 1 | 2 | 1 | 1 | 1 | 0 | 0 | 0 |
| **Patella** | 0 | 1 | 0 | 0 | 0 | 0 | 0 | 0 |
| **Tibia** | 0 | 9 | 0 | 0 | 0 | 0 | 0 | 0 |
| **Fibula** | 0 | 2 | 0 | 0 | 0 | 0 | 0 | 0 |
| **Tarsals** | 3 | 1 | 0 | 2 | 1 | 1 | 0 | 0 |
| **Foot phalanges** | 2 | 4 | 5 | 8 | 7 | 3 | 1 | 0 |
| **Dentition** | 31 | 0 | 9 | 6 | 8 | 10 | 4 | 0 |
| **Other** | 1 | 0 | 0 | 1 | 0 | 0 | 0 | 1 |
| ***SUBTOTAL*** | **54** | **70** | **116** | **80** | **109** | **77** | **24** | **1** |
| **Number measurable specimens** | **3** | **10** | **7** | **6** | **11** | **7** | **5** | **0** |
| **Unidentifiable fragments** | 7 | 39 | 204 | 78 | 95 | 54 | 54 | 0 |
| ***TOTAL*** | **61** | **109** | **320** | **158** | **204** | **131** | **78** | **1** |
| ***GRAND TOTAL*** |  |  |  |  |  |  |  | ***1062*** |

**Supplementary Data 2** - A summary table of the NISP (number of individual specimens) collected from Ucheliung Cave as well as a complete list of all specimens found in Ucheliung cave indicating whether the specimens were measurable as well as those specimens used for AMS and DNA analysis.

| **Ucheliung Cave** |  |  |  |  |  |
| --- | --- | --- | --- | --- | --- |
| **Museum Specimen number** | **Field allocation number** | **Description** | **LEVEL** | **COMMENTS** | **Measurements taken?** |
| **B:OR-14:8-001** | **FS-06-01** | Frontal fragment | "Cave extension" |  | **Y** |
| **B:OR-14:8-002** | **FS-06-02** | Frontal and Parietal fragment | "Cave extension" |  |  |
| **B:OR-14:8-003** | **FS-06-03** | Left proximal. Tibia | "Cave extension" |  | **Y** |
| **B:OR-14:8-004** | **FS-06-04** | R Femoral Diaphysis | "Cave extension" |  |  |
| **B:OR-14:8-005** | **FS-06-05** | Left Temporal fragment | "Cave extension" |  |  |
| **B:OR-14:8-006** | **FS-06-06** | 1st Metatarsal right | "Cave extension" |  | **Y** |
| **B:OR-14:8-007** | **FS-06-07** | Right Parietal 2x pieces | "Cave extension" |  |  |
| **B:OR-14:8-008** | **FS-06-08** | Right 4th Metatarsal | "Cave extension" |  | **Y** |
| **B:OR-14:8-009** | **FS-06-09** | Refit with FS-06-02 | "Cave extension" |  |  |
| **B:OR-14:8-010** | **FS-06-10** | Cranial vault fragment | "Cave extension" |  |  |
| **B:OR-14:8-011** | **FS-06-11** | Right Distal Tibia | "Cave extension" |  | **Y** |
| **B:OR-14:8-012** | **FS-06-12** | Refit with FS-06-07 | "Cave extension" |  |  |
| **B:OR-14:8-013** | **FS-06-13** | Left proximal Diaphysis of femur | "Cave extension" |  |  |
| **B:OR-14:8-014** | **FS-06-14** | Tibia Shaft fragment | "Cave extension" |  |  |
| **B:OR-14:8-015** | **FS-06-15** | Frontal fragment. with R orbit | "Cave extension" |  |  |
| **B:OR-14:8-016** | **FS-06-16** | Distal femur fragment | "Cave extension" |  |  |
| **B:OR-14:8-017** | **FS-06-17** | Left tibial shaft fragment | "Cave extension" |  |  |
| **B:OR-14:8-018** | **FS-06-18** | Refit with FS-06-10 | "Cave extension" |  |  |
| **B:OR-14:8-019** | **FS-06-19** | Tibial shaft fragment | "Cave extension" |  |  |
| **B:OR-14:8-020** | **FS-06-20** | Refit with FS-06-25 | "Cave extension" |  |  |
| **B:OR-14:8-021** | **FS-06-21** | Cranial vault fragment | "Cave extension" |  |  |
| **B:OR-14:8-022** | **FS-06-22** | Right distal Radius | "Cave extension" |  | **Y** |
| **B:OR-14:8-023** | **FS-06-23** | Left Temporal fragment | "Cave extension" |  |  |
| **B:OR-14:8-024** | **FS-06-24** | Right distal Tibia | "Cave extension" |  |  |
| **B:OR-14:8-025** | **FS-06-25** | Left Parietal fragment 4x pieces | "Cave extension" |  |  |
| **B:OR-14:8-026** | **FS-06-26** | Left Parietal fragment | "Cave extension" |  |  |
| **B:OR-14:8-027** | **FS-06-27** | Refit with FS -06-25 | "Cave extension" |  |  |
| **B:OR-14:8-028** | **FS-06-28** | Refit with FS-06-02 | "Cave extension" |  |  |
| **B:OR-14:8-029** | **FS-06-29** | Occipital fragment | "Cave extension" |  |  |
| **B:OR-14:8-030** | **FS-06-30** | Cranial vault fragment | "Cave extension" |  |  |
| **B:OR-14:8-031** | **FS-06-31** | Right Parietal fragment | "Cave extension" |  |  |
| **B:OR-14:8-032** | **FS-06-32** | Cranial vault fragment | "Cave extension" |  |  |
| **B:OR-14:8-033** | **FS-06-33** | Occipital fragment | "Cave extension" |  |  |
| **B:OR-14:8-034** | **FS-06-34** | Left Temporal fragment | "Cave extension" |  |  |
| **B:OR-14:8-035** | **FS-06-35** | Cranial vault fragment | "Cave extension" |  |  |
| **B:OR-14:8-036** | **FS-06-36** | Long bone fragment | "Cave extension" |  |  |
| **B:OR-14:8-037** | **FS-06-37** | Cranial vault fragment | "Cave extension" |  |  |
| **B:OR-14:8-038** | **FS-06-38** | Refit with FS-06-25 | "Cave extension" |  |  |
| **B:OR-14:8-039** | **FS-06-39** | Cranial vault fragment | "Cave extension" |  |  |
| **B:OR-14:8-040** | **FS-06-40** | Cranial vault fragment | "Cave extension" |  |  |
| **B:OR-14:8-041** | **FS-06-41** | Refit with FS-06-30 | "Cave extension" |  |  |
| **B:OR-14:8-042** | **FS-06-42** | Long bone fragment | "Cave extension" |  |  |
| **B:OR-14:8-043** | **FS-06-43** | Left Tibial diaphysis | "Cave extension" |  | **Y** |
| **B:OR-14:8-044** | **FS-06-44** | Os coxa | "Cave extension" | Juvenile |  |
| **B:OR-14:8-045** | **FS-06-45** | Long bone fragment | "Cave extension" |  |  |
| **B:OR-14:8-046** | **FS-06-46** | Long bone fragment | "Cave extension" |  |  |
| **B:OR-14:8-047** | **FS-06-47** | Long bone fragment | "Cave extension" |  |  |
| **B:OR-14:8-048** | **FS-06-48** | Long bone fragment | "Cave extension" |  |  |
| **B:OR-14:8-049** | **FS-06-49** | Right distal Fibula | "Cave extension" |  | **Y** |
| **B:OR-14:8-050** | **FS-06-50** | Long bone fragment | "Cave extension" |  |  |
| **B:OR-14:8-051** | **FS-06-51** | L Distal fibula | "Cave extension" |  | **Y** |
| **B:OR-14:8-052** | **FS-06-52** | Long bone fragment | "Cave extension" |  |  |
| **B:OR-14:8-053** | **FS-06-53** | Long bone fragment | "Cave extension" |  |  |
| **B:OR-14:8-054** | **FS-06-54** | Long bone fragment | "Cave extension" |  |  |
| **B:OR-14:8-055** | **FS-06-55** | Left distal tibia | "Cave extension" |  |  |
| **B:OR-14:8-056** | **FS-06-56** | Long bone fragment | "Cave extension" |  |  |
| **B:OR-14:8-057** | **FS-06-57** | Rib Fragment | "Cave extension" |  |  |
| **B:OR-14:8-058** | **FS-06-58** | Long bone fragment | "Cave extension" |  |  |
| **B:OR-14:8-059** | **FS-06-59** | Long bone fragment | "Cave extension" |  |  |
| **B:OR-14:8-060** | **FS-06-60** | Left Patella | "Cave extension" |  | **Y** |
| **B:OR-14:8-061** | **FS-06-61** | Humeral head fragment | "Cave extension" |  |  |
| **B:OR-14:8-062** | **FS-06-62** | Cranial vault fragment | "Cave extension" |  |  |
| **B:OR-14:8-063** | **FS-06-63** | Long bone fragment | "Cave extension" |  |  |
| **B:OR-14:8-064** | **FS-06-64** | Long bone fragment | "Cave extension" |  |  |
| **B:OR-14:8-065** | **FS-06-65** | Occipital fragment | "Cave extension" |  |  |
| **B:OR-14:8-066** | **FS-06-66** | Right Calcaneus fragment | "Cave extension" |  |  |
| **B:OR-14:8-067** | **FS-06-67** | Right sphenoid fragment | "Cave extension" |  |  |
| **B:OR-14:8-068** | **FS-06-68** | Cranial vault fragment | "Cave extension" |  |  |
| **B:OR-14:8-069** | **FS-06-69** | Long bone fragment | "Cave extension" |  |  |
| **B:OR-14:8-070** | **FS-06-70** | Sphenoid fragment | "Cave extension" |  |  |
| **B:OR-14:8-071** | **FS-06-71** | Long bone fragment | "Cave extension" |  |  |
| **B:OR-14:8-072** | **FS-06-72** | Long bone fragment | "Cave extension" |  |  |
| **B:OR-14:8-073** | **FS-06-73** | Cranial vault fragment | "Cave extension" |  |  |
| **B:OR-14:8-074** | **FS-06-74** | Temporal fragment | "Cave extension" |  |  |
| **B:OR-14:8-075** | **FS-06-75** | Cranial vault fragment | "Cave extension" |  |  |
| **B:OR-14:8-076** | **FS-06-76** | Long bone fragment | "Cave extension" |  |  |
| **B:OR-14:8-077** | **FS-06-77** | Cranial vault fragment | "Cave extension" |  |  |
| **B:OR-14:8-078** | **FS-06-78** | Cranial vault fragment | "Cave extension" |  |  |
| **B:OR-14:8-079** | **FS-06-79** | Non- identifiable | "Cave extension" |  |  |
| **B:OR-14:8-080** | **FS-06-80** | Cranial vault fragment | "Cave extension" |  |  |
| **B:OR-14:8-081** | **FS-06-81** | Cranial vault fragment | "Cave extension" |  |  |
| **B:OR-14:8-082** | **FS-06-82** | Occipital. Process of the Thoracic vertebrae | "Cave extension" |  |  |
| **B:OR-14:8-083** | **FS-06-83** | Long bone fragment | "Cave extension" |  |  |
| **B:OR-14:8-084** | **FS-06-84** | Long bone fragment | "Cave extension" |  |  |
| **B:OR-14:8-085** | **FS-06-85** | Long bone fragment | "Cave extension" |  |  |
| **B:OR-14:8-086** | **FS-06-86** | Non- identifiable | "Cave extension" |  |  |
| **B:OR-14:8-087** | **FS-06-87** | Non- identifiable | "Cave extension" |  |  |
| **B:OR-14:8-088** | **FS-06-88** | Non- identifiable | "Cave extension" |  |  |
| **B:OR-14:8-089** | **FS-06-89** | Non- identifiable | "Cave extension" |  |  |
| **B:OR-14:8-090** | **FS-06-90** | Metatarsal fragment | Inner Chamber |  |  |
| **B:OR-14:8-091** | **FS-06-91** | Cranial vault fragment | Inner Chamber |  |  |
| **B:OR-14:8-092** | **FS-06-92** | Right Temporal fragment | "Cave extension" |  |  |
| **B:OR-14:8-093** | **FS-06-93** | Cranial vault fragment | "Cave extension" |  |  |
| **B:OR-14:8-094** | **FS-06-94** | Cranial vault fragment | "Cave extension" |  |  |
| **B:OR-14:8-095** | **FS-06-95** | Cranial vault fragment | "Cave extension" |  |  |
| **B:OR-14:8-096** | **FS-06-96** | Long bone fragment | "Cave extension" |  |  |
| **B:OR-14:8-098** | **FS-06-98** | R proximal 1st Metatarsal | "Cave extension" |  | **Y** |
| **B:OR-14:8-100** | **FS-06-100** | Long bone fragment | Surface |  |  |
| **B:OR-14:8-101** | **FS-06-101** | Long bone fragment | Surface |  |  |
| **B:OR-14:8-102** | **FS-06-102** | Cranial vault fragment | Surface |  |  |
| **B:OR-14:8-103** | **FS-06-103** | Non- identifiable | Surface |  |  |
| **B:OR-14:8-104** | **FS-06-104** | Rib Fragment | Surface |  |  |
| **B:OR-14:8-105** | **FS-06-105** | Long bone fragment | Surface |  |  |
| **B:OR-14:8-106** | **FS-06-106** | Cranial vault fragment | Surface |  |  |
| **B:OR-14:8-107** | **FS-06-107** | Incisor | Surface | DNA |  |
| **B:OR-14:8-108** | **FS-06-108** | Left mandible | Surface | DNA | **Y** |
| **B:OR-14:8-109** | **FS-06-109** | Right Talus | Surface |  | **Y** |
| **B:OR-14:8-110** | **FS-06-110** | Long bone fragment | Surface |  |  |
| **B:OR-14:8-111** | **FS-06-111** | Long bone fragment | Surface |  |  |
| **B:OR-14:8-112** | **FS-06-112** | Cranial vault fragment | Surface |  |  |
| **B:OR-14:8-113** | **FS-06-113** | Vertebral Fragment | Surface |  |  |
| **B:OR-14:8-114** | **FS-06-114** | Rib Fragment | Surface |  |  |
| **B:OR-14:8-115** | **FS-06-115** | Rib Fragment | Surface |  |  |
| **B:OR-14:8-116** | **FS-06-116** | Pedal Phalanx | Surface |  |  |
| **B:OR-14:8-117** | **FS-06-117** | Femoral diaphyseal fragment | Surface |  |  |
| **B:OR-14:8-118** | **FS-06-118** | Premolar | Surface | DNA |  |
| **B:OR-14:8-119** | **FS-06-119** | RP_4_ | Surface |  | **Y** |
| **B:OR-14:8-120** | **FS-06-120** | Incisor | Surface | DNA |  |
| **B:OR-14:8-121** | **FS-06-121** | RM_2_ | Surface | Root fused | **Y** |
| **B:OR-14:8-122** | **FS-06-122** | Right mandible | Surface | Contains the following teeth for which measurements were taken: RM_1/2_ | **Y** |
| **B:OR-14:8-123** | **FS-06-123** | LM_1_ | Surface |  | **Y** |
| **B:OR-14:8-125** | **FS-06-125** | Fragment R Scapula | Surface |  |  |
| **B:OR-14:8-126** | **FS-06-126** | LC^1^ | Surface |  | **Y** |
| **B:OR-14:8-127** | **FS-06-127** | Pedal Phalanx- distal | Surface |  |  |
| **B:OR-14:8-128** | **FS-06-128** | I^1^ | Surface | partial crown |  |
| **B:OR-14:8-130** | **FS-06-130** | LM^2^ | Surface |  | **Y** |
| **B:OR-14:8-132** | **FS-06-132** | Right cuboid | Surface |  |  |
| **B:OR-14:8-134** | **FS-06-134** | Scapula fragment | Surface |  |  |
| **B:OR-14:8-135** | **FS-06-135** | Long bone fragment | Surface |  |  |
| **B:OR-14:8-136** | **FS-06-136** | RP_3_ | Surface |  | **Y** |
| **B:OR-14:8-138** | **FS-06-138** | Right Navicular fragment | Surface |  |  |
| **B:OR-14:8-139** | **FS-06-139** | RI^1^ | Surface |  | **Y** |
| **B:OR-14:8-140** | **FS-06-140** | LI^2^ | Surface |  | **Y** |
| **B:OR-14:8-141** | **FS-06-141** | Lam and transverse process of thoracic vertebra | Surface |  |  |
| **B:OR-14:8-142** | **FS-06-142** | LP_3_ | Surface |  | **Y** |
| **B:OR-14:8-143** | **FS-06-143** | Right zygomatic | Surface |  |  |
| **B:OR-14:8-146** | **FS-06-146** | RI_1_ | Surface |  | **Y** |
| **B:OR-14:8-147** | **FS-06-147** | LP_3_ | Surface |  | **Y** |
| **B:OR-14:8-149** | **FS-06-149** | Artifacts | Surface |  |  |
| **B:OR-14:8-151** | **FS-06-151** | Juvenile rib | Surface |  |  |
| **B:OR-14:8-153** | **FS-06-153** | LC^1^ | Surface |  | **Y** |
| **B:OR-14:8-154** | **FS-06-154** | LM_1_ | Surface |  | **Y** |
| **B:OR-14:8-155** | **FS-06-155** | RI^2^ | Surface |  | **Y** |
| **B:OR-14:8-156** | **FS-06-156** | LI_1_ | Surface |  | **Y** |
| **B:OR-14:8-158** | **FS-06-158** | LP^3^ | Surface |  | **Y** |
| **B:OR-14:8-161** | **FS-06-161** | RP_3_ | Surface |  | **Y** |
| **B:OR-14:8-162** | **FS-06-162** | Cranial vault fragment | Surface |  |  |
| **B:OR-14:8-163A** | **FS-06-163A** | LP_3_ | Surface |  | **Y** |
| **B:OR-14:8-163B** | **FS-06-163B** | RM^1^ | Surface |  | **Y** |
| **B:OR-14:8-164** | **FS-06-164** | LM_3_ | Surface |  | **Y** |
| **B:OR-14:8-165** | **FS-06-165** | RI^1^ | Surface | Paired | **Y** |
|  | **FS-06-165** | LI^1^ | Surface |  | **Y** |
| **B:OR-14:8-166** | **FS-06-166** | Bone Fragment | L2 | DNA |  |
| **B:OR-14:8-167** | **FS-06-167** | Bone Fragment | L2 | DNA |  |
| **B:OR-14:8-168** | **FS-06-168** | Bone Fragment | L2 | DNA |  |
| **B:OR-14:8-169** | **FS-06-169** | Bone Fragment | L2 | DNA |  |
| **B:OR-14:8-170** | **FS-06-170** | LI^2^ | L2 |  | **Y** |
| **B:OR-14:8-171** | **FS-06-171** | Bone Fragment | L2 | DNA |  |
| **B:OR-14:8-172** | **FS-06-172** | RC^1^ | Surface |  | **Y** |
| **B:OR-14:8-173** | **FS-06-173** | RI_2_ | Surface |  | **Y** |
| **B:OR-14:8-174** | **FS-06-174** | **Infant burial**: | unknown provenience | 7x partial Verte centra 10 x Verte Neural Arch elements 14 x rib frag 3x CVF 3x NID Aslo found with this specimen, 1 x Rm (max) 1 xCanine adult P^3+4^ |  |
| **B:OR-14:8-175** | **FS-06-175** | fragment Juvenile maxilla | Surface | Includes: Rm^1^, RC^1^, P^3+4^ | **Y** |
| **B:OR-14:8-176** | **FS-06-175A** | RP^3^ | Surface |  | **Y** |
| **B:OR-14:8-177** | **FS-06-175B** | RP^4^ | Surface |  | **Y** |
| **B:OR-14:8-178** | **FS-06-175C** | RC^1^ | Surface |  | **Y** |
| **B:OR-14:8-179** | **FS-06-200** | Cranial vault fragment | L1 |  |  |
| **B:OR-14:8-180** | **FS-06-201** | Mandibular fragment | L1 |  |  |
| **B:OR-14:8-181** | **FS-06-202** | Cranial vault fragment | L1 |  |  |
| **B:OR-14:8-182** | **FS-06-203** | Cranial vault fragment | L1 |  |  |
| **B:OR-14:8-183** | **FS-06-204** | Long bone fragment | L1 |  |  |
| **B:OR-14:8-184** | **FS-06-205** | Maxillary fragment | L1 |  |  |
| **B:OR-14:8-185** | **FS-06-206** | Long bone fragment | L1 |  |  |
| **B:OR-14:8-186** | **FS-06-207** | Long bone fragment | L1 |  |  |
| **B:OR-14:8-187** | **FS-06-208** | Long bone fragment | L1 |  |  |
| **B:OR-14:8-188** | **FS-06-209** | Long bone fragment | L1 |  |  |
| **B:OR-14:8-189** | **FS-06-210** | Long bone fragment | L1 |  |  |
| **B:OR-14:8-190** | **FS-06-211** | Vertebral Fragment | L1 |  |  |
| **B:OR-14:8-191** | **FS-06-212** | Long bone fragment | L1 |  |  |
| **B:OR-14:8-192** | **FS-06-213** | Non- identifiable | L1 |  |  |
| **B:OR-14:8-193** | **FS-06-214** | Vertebral Fragment | L1 |  |  |
| **B:OR-14:8-194** | **FS-06-215** | Non- identifiable | L1 |  |  |
| **B:OR-14:8-195** | **FS-06-216** | Non- identifiable | L1 |  |  |
| **B:OR-14:8-196** | **FS-06-217** | Long bone fragment | L1 |  |  |
| **B:OR-14:8-197** | **FS-06-218** | Long bone fragment | L1 |  |  |
| **B:OR-14:8-198** | **FS-06-219** | Cranial vault fragment | L1 |  |  |
| **B:OR-14:8-199** | **FS-06-220** | Cranial vault fragment | L1 |  |  |
| **B:OR-14:8-200** | **FS-06-221** | Long bone fragment | L1 |  |  |
| **B:OR-14:8-201** | **FS-06-222** | Cranial vault fragment | L1 |  |  |
| **B:OR-14:8-202** | **FS-06-223** | Cranial vault fragment | L1 |  |  |
| **B:OR-14:8-203** | **FS-06-224** | Non- identifiable | L1 |  |  |
| **B:OR-14:8-204** | **FS-06-225** | Cranial vault fragment | L1 |  |  |
| **B:OR-14:8-205** | **FS-06-226** | Long bone fragment | L1 |  |  |
| **B:OR-14:8-206** | **FS-06-227** | Long bone fragment | L1 |  |  |
| **B:OR-14:8-207** | **FS-06-228** | RI_2_ | L1 |  | **Y** |
| **B:OR-14:8-208** | **FS-06-229** | Long bone fragment | L1 |  |  |
| **B:OR-14:8-209** | **FS-06-230** | Long bone fragment | L1 |  |  |
| **B:OR-14:8-210** | **FS-06-231** | Long bone fragment | L1 |  |  |
| **B:OR-14:8-211** | **FS-06-232** | Non- identifiable | L1 |  |  |
| **B:OR-14:8-212** | **FS-06-233** | Long bone fragment | L1 |  |  |
| **B:OR-14:8-213** | **FS-06-234** | Non- identifiable | L1 |  |  |
| **B:OR-14:8-214** | **FS-06-235** | Non- identifiable | L1 |  |  |
| **B:OR-14:8-215** | **FS-06-236** | Juvenile rib | L1 |  |  |
| **B:OR-14:8-216** | **FS-06-237** | Non- identifiable | L1 |  |  |
| **B:OR-14:8-217** | **FS-06-238** | Long bone fragment | L1 |  |  |
| **B:OR-14:8-218** | **FS-06-239** | Non- identifiable | L1 |  |  |
| **B:OR-14:8-219** | **FS-06-240** | Rib Fragment | L1 |  |  |
| **B:OR-14:8-220** | **FS-06-241** | Long bone fragment | L1 |  |  |
| **B:OR-14:8-221** | **FS-06-242** | Long bone fragment | L1 |  |  |
| **B:OR-14:8-222** | **FS-06-243** | Non- identifiable | L1 |  |  |
| **B:OR-14:8-223** | **FS-06-244** | Non- identifiable | L1 |  |  |
| **B:OR-14:8-224** | **FS-06-245** | Tooth fragment | L1 |  |  |
| **B:OR-14:8-225** | **FS-06-246** | Long bone fragment | L1 |  |  |
| **B:OR-14:8-226** | **FS-06-247** | Cranial vault fragment | L1 |  |  |
| **B:OR-14:8-227** | **FS-06-248** | Non- identifiable | L1 |  |  |
| **B:OR-14:8-228** | **FS-06-249** | Cranial vault fragment | L1 |  |  |
| **B:OR-14:8-229** | **FS-06-250** | Non- identifiable | L1 |  |  |
| **B:OR-14:8-230** | **FS-06-251** | Non- identifiable | L1 |  |  |
| **B:OR-14:8-231** | **FS-06-252** | Non- identifiable | L1 |  |  |
| **B:OR-14:8-232** | **FS-06-253** | Non- identifiable | L1 |  |  |
| **B:OR-14:8-233** | **FS-06-254** | Non- identifiable | L1 |  |  |
| **B:OR-14:8-234** | **FS-06-255** | Tooth crown fragment | L1 |  |  |
| **B:OR-14:8-235** | **FS-06-256** | Non- identifiable | L1 |  |  |
| **B:OR-14:8-236** | **FS-06-257** | Non- identifiable | L1 |  |  |
| **B:OR-14:8-237** | **FS-06-258** | Non- identifiable | L1 |  |  |
| **B:OR-14:8-238** | **FS-06-259** | Non- identifiable | L1 |  |  |
| **B:OR-14:8-239** | **FS-06-260** | Non- identifiable | L1 |  |  |
| **B:OR-14:8-240** | **FS-06-261** | Non- identifiable | L1 |  |  |
| **B:OR-14:8-241** | **FS-06-262** | Non- identifiable | L1 |  |  |
| **B:OR-14:8-242** | **FS-06-263** | Non- identifiable | L1 |  |  |
| **B:OR-14:8-243** | **FS-06-264** | Non- identifiable | L1 |  |  |
| **B:OR-14:8-244** | **FS-06-265** | Non- identifiable | L1 |  |  |
| **B:OR-14:8-245** | **FS-06-266** | Non- identifiable | L1 |  |  |
| **B:OR-14:8-246** | **FS-06-267** | Non- identifiable | L1 |  |  |
| **B:OR-14:8-247** | **FS-06-268** | Non- identifiable | L1 |  |  |
| **B:OR-14:8-248** | **FS-06-269** | Non- identifiable | L1 |  |  |
| **B:OR-14:8-249** | **FS-06-270** | Non- identifiable | L1 |  |  |
| **B:OR-14:8-250** | **FS-06-271** | Non- identifiable | L1 |  |  |
| **B:OR-14:8-251** | **FS-06-272** | Non- identifiable | L1 |  |  |
| **B:OR-14:8-252** | **FS-06-273** | Non- identifiable | L1 |  |  |
| **B:OR-14:8-253** | **FS-06-274** | Non- identifiable | L1 |  |  |
| **B:OR-14:8-254** | **FS-06-275** | Non- identifiable | L1 |  |  |
| **B:OR-14:8-255** | **FS-06-276** | Non- identifiable | L1 |  |  |
| **B:OR-14:8-256** | **FS-06-277** | Non- identifiable | L1 |  |  |
| **B:OR-14:8-257** | **FS-06-278** | Non- identifiable | L1 |  |  |
| **B:OR-14:8-258** | **FS-06-279** | Non- identifiable | L1 |  |  |
| **B:OR-14:8-259** | **FS-06-280** | Non- identifiable | L1 |  |  |
| **B:OR-14:8-260** | **FS-06-281** | Non- identifiable | L1 |  |  |
| **B:OR-14:8-261** | **FS-06-282** | Non- identifiable | L1 |  |  |
| **B:OR-14:8-262** | **FS-06-283** | Non- identifiable | L1 |  |  |
| **B:OR-14:8-263** | **FS-06-284** | Non- identifiable | L1 |  |  |
| **B:OR-14:8-264** | **FS-06-285** | Non- identifiable | L1 |  |  |
| **B:OR-14:8-265** | **FS-06-286** | Non- identifiable | L1 |  |  |
| **B:OR-14:8-266** | **FS-06-287** | Non- identifiable | L1 |  |  |
| **B:OR-14:8-267** | **FS-06-288** | Non- identifiable | L1 |  |  |
| **B:OR-14:8-268** | **FS-06-289** | Non- identifiable | L1 |  |  |
| **B:OR-14:8-269** | **FS-06-290** | Non- identifiable | L1 |  |  |
| **B:OR-14:8-270** | **FS-06-291** | Non- identifiable | L1 |  |  |
| **B:OR-14:8-271** | **FS-06-292** | Rib Fragment | L1 |  |  |
| **B:OR-14:8-272** | **FS-06-293** | Bone Fragment | L1 | AMS Sample |  |
| **B:OR-14:8-273** | **FS-06-294** | Right 3rd metacarpal (proximal) | L1 |  | **Y** |
| **B:OR-14:8-275** | **FS-06-296** | Vertebral Fragment | L1 |  |  |
| **B:OR-14:8-276** | **FS-06-297** | Left Lunate | L1 |  | **Y** |
| **B:OR-14:8-277** | **FS-06-298** | Right Lunate | L1 |  | **Y** |
| **B:OR-14:8-278** | **FS-06-299** | Rib Fragment | L1 |  |  |
| **B:OR-14:8-279** | **FS-06-300** | Rib Fragment | L1 |  |  |
| **B:OR-14:8-280** | **FS-06-301** | Rib Fragment | L1 |  |  |
| **B:OR-14:8-281** | **FS-06-302** | Long bone fragment | L1 |  |  |
| **B:OR-14:8-282** | **FS-06-303** | Long bone fragment | L1 |  |  |
| **B:OR-14:8-283** | **FS-06-304** | Cranial vault fragment | L1 |  |  |
| **B:OR-14:8-284** | **FS-06-305** | Long bone fragment | L1 |  |  |
| **B:OR-14:8-285** | **FS-06-306** | Long bone fragment | L1 |  |  |
| **B:OR-14:8-286** | **FS-06-307** | Rib Fragment | L1 |  |  |
| **B:OR-14:8-287** | **FS-06-308** | Cranial vault fragment | L1 |  |  |
| **B:OR-14:8-288** | **FS-06-309** | Rib Fragment | L1 |  |  |
| **B:OR-14:8-289** | **FS-06-310** | Manual interm. Phalanx | L1 |  |  |
| **B:OR-14:8-290** | **FS-06-311** | Vertebral Fragment | L1 |  |  |
| **B:OR-14:8-291** | **FS-06-312** | Distal Man. Phalanx | L1 |  |  |
| **B:OR-14:8-292** | **FS-06-313** | Rib Fragment | L1 |  |  |
| **B:OR-14:8-293** | **FS-06-314** | Long bone fragment | L1 |  |  |
| **B:OR-14:8-294** | **FS-06-315** | Long bone fragment | L1 |  |  |
| **B:OR-14:8-295** | **FS-06-316** | Lc^1^ | L1 |  | **Y** |
| **B:OR-14:8-296** | **FS-06-317** | Femoral condyle fragment | L1 |  |  |
| **B:OR-14:8-297** | **FS-06-318** | Rib Fragment | L1 |  |  |
| **B:OR-14:8-298** | **FS-06-319** | Cranial vault fragment | L1 |  |  |
| **B:OR-14:8-299** | **FS-06-320** | Non- identifiable | L1 |  |  |
| **B:OR-14:8-301** | **FS-06-322** | 1st Distal pedal phalanx | L1 |  |  |
| **B:OR-14:8-302** | **FS-06-323** | Vertebral Fragment | L1 |  |  |
| **B:OR-14:8-303** | **FS-06-324** | Long bone fragment | L1 |  |  |
| **B:OR-14:8-304** | **FS-06-325** | Non- identifiable | L1 |  |  |
| **B:OR-14:8-305** | **FS-06-326** | Rib Fragment | L1 |  |  |
| **B:OR-14:8-306** | **FS-06-327** | Rib Fragment | L1 |  |  |
| **B:OR-14:8-307** | **FS-06-328** | Non- identifiable | L1 |  |  |
| **B:OR-14:8-308** | **FS-06-329** | Vertebral Fragment | L1 |  |  |
| **B:OR-14:8-309** | **FS-06-330** | Long bone fragment | L1 |  |  |
| **B:OR-14:8-310** | **FS-06-331** | Rib Fragment | L1 |  |  |
| **B:OR-14:8-311** | **FS-06-332** | Long bone fragment | L1 |  |  |
| **B:OR-14:8-312** | **FS-06-333** | Long bone fragment | L1 |  |  |
| **B:OR-14:8-313** | **FS-06-334** | Left Triquetral | L1 |  | **Y** |
| **B:OR-14:8-314** | **FS-06-335** | Cranial vault fragment | L1 |  |  |
| **B:OR-14:8-315** | **FS-06-336** | Long bone fragment | L1 |  |  |
| **B:OR-14:8-316** | **FS-06-337** | Long bone fragment | L1 |  |  |
| **B:OR-14:8-317** | **FS-06-338** | Rib Fragment | L1 |  |  |
| **B:OR-14:8-318** | **FS-06-339** | Rib Fragment | L1 |  |  |
| **B:OR-14:8-319** | **FS-06-340** | Long bone fragment | L1 |  |  |
| **B:OR-14:8-320** | **FS-06-341** | Long bone fragment | L1 |  |  |
| **B:OR-14:8-321** | **FS-06-342** | Long bone fragment | L1 |  |  |
| **B:OR-14:8-322** | **FS-06-343** | Long bone fragment | L1 |  |  |
| **B:OR-14:8-323** | **FS-06-344** | Scapula fragment | L1 |  |  |
| **B:OR-14:8-324** | **FS-06-345** | Long bone fragment | L1 |  |  |
| **B:OR-14:8-325** | **FS-06-346** | Long bone fragment | L1 |  |  |
| **B:OR-14:8-326** | **FS-06-347** | Vertebral Fragment | L1 |  |  |
| **B:OR-14:8-327** | **FS-06-348** | Long bone fragment | L1 |  |  |
| **B:OR-14:8-329** | **FS-06-350** | Cranial vault fragment | L1 |  |  |
| **B:OR-14:8-330** | **FS-06-351** | Cranial vault fragment | L1 |  |  |
| **B:OR-14:8-331** | **FS-06-352** | Non- identifiable | L1 |  |  |
| **B:OR-14:8-332** | **FS-06-353** | Non- identifiable | L1 |  |  |
| **B:OR-14:8-333** | **FS-06-354** | proximal. Manual Phalanx | L1 | Juvenile |  |
| **B:OR-14:8-334** | **FS-06-355** | Long bone fragment | L1 |  |  |
| **B:OR-14:8-335** | **FS-06-356** | Non- identifiable | L1 |  |  |
| **B:OR-14:8-336** | **FS-06-357** | Non- identifiable | L1 |  |  |
| **B:OR-14:8-337** | **FS-06-358** | Non- identifiable | L1 |  |  |
| **B:OR-14:8-338** | **FS-06-359** | Long bone fragment | L1 |  |  |
| **B:OR-14:8-339** | **FS-06-360** | Long bone fragment | L1 |  |  |
| **B:OR-14:8-340** | **FS-06-361** | Long bone fragment | L1 |  |  |
| **B:OR-14:8-341** | **FS-06-362** | Long bone fragment | L1 |  |  |
| **B:OR-14:8-342** | **FS-06-363** | Rib Fragment | L1 |  |  |
| **B:OR-14:8-343** | **FS-06-364** | Rib Fragment | L1 |  |  |
| **B:OR-14:8-344** | **FS-06-365** | Non- identifiable | L1 |  |  |
| **B:OR-14:8-345** | **FS-06-366** | Non- identifiable | L1 |  |  |
| **B:OR-14:8-346** | **FS-06-367** | Non- identifiable | L1 |  |  |
| **B:OR-14:8-347** | **FS-06-368** | Non- identifiable | L1 |  |  |
| **B:OR-14:8-348** | **FS-06-369** | Non- identifiable | L1 |  |  |
| **B:OR-14:8-349** | **FS-06-370** | Non- identifiable | L1 |  |  |
| **B:OR-14:8-350** | **FS-06-371** | Non- identifiable | L1 |  |  |
| **B:OR-14:8-351** | **FS-06-372** | Non- identifiable | L1 |  |  |
| **B:OR-14:8-352** | **FS-06-373** | Non- identifiable | L1 |  |  |
| **B:OR-14:8-353** | **FS-06-374** | Non- identifiable | L1 |  |  |
| **B:OR-14:8-354** | **FS-06-375** | Non- identifiable | L1 |  |  |
| **B:OR-14:8-355** | **FS-06-376** | Non- identifiable | L1 |  |  |
| **B:OR-14:8-356** | **FS-06-377** | Non- identifiable | L1 |  |  |
| **B:OR-14:8-357** | **FS-06-378** | Non- identifiable | L1 |  |  |
| **B:OR-14:8-358** | **FS-06-379** | Non- identifiable | L1 |  |  |
| **B:OR-14:8-359** | **FS-06-380** | Non- identifiable | L1 |  |  |
| **B:OR-14:8-360** | **FS-06-381** | Non- identifiable | L1 |  |  |
| **B:OR-14:8-361** | **FS-06-382** | Non- identifiable | L1 |  |  |
| **B:OR-14:8-362** | **FS-06-383** | Non- identifiable | L1 |  |  |
| **B:OR-14:8-363** | **FS-06-384** | Non- identifiable | L1 |  |  |
| **B:OR-14:8-364** | **FS-06-385** | Left proximal Rib | L1 |  |  |
| **B:OR-14:8-365** | **FS-06-386** | Long bone fragment | L1 |  |  |
| **B:OR-14:8-366** | **FS-06-387** | Radial Diaphysis | L1 |  |  |
| **B:OR-14:8-367** | **FS-06-388** | ? | L1 | AMS Sample |  |
| **B:OR-14:8-368** | **FS-06-389** | Juvenile Rib Fragment | L1 |  |  |
| **B:OR-14:8-369** | **FS-06-390** | Coccyx element 1 | L1 |  |  |
| **B:OR-14:8-370** | **FS-06-391** | Rib Fragment | L1 |  |  |
| **B:OR-14:8-371** | **FS-06-392** | Long bone fragment | L1 |  |  |
| **B:OR-14:8-374** | **FS-06-395** | Scapula fragment | L1 |  |  |
| **B:OR-14:8-375** | **FS-06-396** | Cranial vault fragment | L1 |  |  |
| **B:OR-14:8-376** | **FS-06-397** | Long bone fragment | L1 |  |  |
| **B:OR-14:8-377** | **FS-06-398** | Cranial vault fragment | L1 |  |  |
| **B:OR-14:8-378** | **FS-06-399** | Thoracic vertebral fragment | L1 |  |  |
| **B:OR-14:8-379** | **FS-06-400** | Distal Metatarsal | L1 |  |  |
| **B:OR-14:8-380** | **FS-06-401** | Bone Fragment | L1 | AMS Sample |  |
| **B:OR-14:8-381** | **FS-06-402** | Bone Fragment | L1 | AMS Sample |  |
| **B:OR-14:8-382** | **FS-06-403** | Long bone fragment | L1 |  |  |
| **B:OR-14:8-383** | **FS-06-404** | Long bone fragment | L1 |  |  |
| **B:OR-14:8-384** | **FS-06-405** | Long bone fragment | L1 |  |  |
| **B:OR-14:8-385** | **FS-06-406** | Cranial vault fragment | L1 |  |  |
| **B:OR-14:8-386** | **FS-06-407** | Long bone fragment | L1 |  |  |
| **B:OR-14:8-387** | **FS-06-408** | Rib Fragment | L1 |  |  |
| **B:OR-14:8-388** | **FS-06-409** | Juvenile. Metacarpal shaft | L1 |  |  |
| **B:OR-14:8-389** | **FS-06-410** | Long bone fragment | L1 |  |  |
| **B:OR-14:8-390** | **FS-06-411** | Long bone fragment | L1 |  |  |
| **B:OR-14:8-391** | **FS-06-412** | Left Hammate | L1 |  |  |
| **B:OR-14:8-392** | **FS-06-413** | Mandibular fragment | L1 |  |  |
| **B:OR-14:8-393** | **FS-06-414** | Long bone fragment | L1 |  |  |
| **B:OR-14:8-394** | **FS-06-415** | Cranial vault fragment | L1 |  |  |
| **B:OR-14:8-395** | **FS-06-416** | Bone Fragment | L1 | AMS Sample |  |
| **B:OR-14:8-396** | **FS-06-417** | Cranial vault fragment | L1 |  |  |
| **B:OR-14:8-397** | **FS-06-418** | Rib Fragment | L1 |  |  |
| **B:OR-14:8-398** | **FS-06-419** | Long bone fragment | L1 |  |  |
| **B:OR-14:8-399** | **FS-06-420** | Long bone fragment | L1 |  |  |
| **B:OR-14:8-400** | **FS-06-421** | Long bone fragment | L1 |  |  |
| **B:OR-14:8-401** | **FS-06-422** | proximal Pedal Phalanx | L1 |  |  |
| **B:OR-14:8-402** | **FS-06-423** | Right Trapezoid | L1 |  |  |
| **B:OR-14:8-403** | **FS-06-424** | Left Trapezoid | L1 |  |  |
| **B:OR-14:8-404** | **FS-06-424B** | LI_2_ | L1 |  | **Y** |
| **B:OR-14:8-405** | **FS-06-425** | Rib Fragment | L1 |  |  |
| **B:OR-14:8-406** | **FS-06-426** | Cranial vault fragment | L1 |  |  |
| **B:OR-14:8-407** | **FS-06-427** | Vertebral Fragment | L1 |  |  |
| **B:OR-14:8-408** | **FS-06-428** | Juvenile vertebral fragment | L1 |  |  |
| **B:OR-14:8-409** | **FS-06-429** | Cranial vault fragment | L1 |  |  |
| **B:OR-14:8-410** | **FS-06-430** | Long bone fragment | L1 |  |  |
| **B:OR-14:8-411** | **FS-06-431** | Cranial vault fragment | L1 |  |  |
| **B:OR-14:8-412** | **FS-06-432** | Long bone fragment | L1 |  |  |
| **B:OR-14:8-413** | **FS-06-433** | Vertebral Fragment | L1 |  |  |
| **B:OR-14:8-414** | **FS-06-434** | Juvenile. Long Bone diaphyseal fragment | L1 |  |  |
| **B:OR-14:8-415** | **FS-06-435** | Long bone fragment | L1 |  |  |
| **B:OR-14:8-416** | **FS-06-436** | Long bone fragment | L1 |  |  |
| **B:OR-14:8-417** | **FS-06-437** | Cranial vault fragment | L1 |  |  |
| **B:OR-14:8-418** | **FS-06-438** | Long bone fragment | L1 |  |  |
| **B:OR-14:8-419** | **FS-06-439** | Long bone fragment | L1 |  |  |
| **B:OR-14:8-420** | **FS-06-440** | Long bone fragment | L1 |  |  |
| **B:OR-14:8-421** | **FS-06-441** | Long bone fragment | L1 |  |  |
| **B:OR-14:8-422** | **FS-06-442** | Rib Fragment | L1 |  |  |
| **B:OR-14:8-423** | **FS-06-443** | Cranial vault fragment | L1 |  |  |
| **B:OR-14:8-424** | **FS-06-444** | Rib Fragment | L1 |  |  |
| **B:OR-14:8-425** | **FS-06-445** | Rib Fragment | L1 |  |  |
| **B:OR-14:8-426** | **FS-06-446** | Cranial vault fragment | L1 |  |  |
| **B:OR-14:8-427** | **FS-06-447** | Cranial vault fragment | L1 |  |  |
| **B:OR-14:8-428** | **FS-06-448** | Non- identifiable | L1 |  |  |
| **B:OR-14:8-429** | **FS-06-449** | Long bone fragment | L1 |  |  |
| **B:OR-14:8-430** | **FS-06-450** | Long bone fragment | L1 |  |  |
| **B:OR-14:8-431** | **FS-06-451** | Phalanx | L1 |  |  |
| **B:OR-14:8-432** | **FS-06-452** | Long bone fragment | L1 |  |  |
| **B:OR-14:8-433** | **FS-06-453** | Long bone fragment | L1 |  |  |
| **B:OR-14:8-434** | **FS-06-454** | Phalanx | L1 |  |  |
| **B:OR-14:8-435** | **FS-06-455** | Phalanx | L1 |  |  |
| **B:OR-14:8-436** | **FS-06-456** | Long bone fragment | L1 |  |  |
| **B:OR-14:8-437** | **FS-06-457** | Long bone fragment | L1 |  |  |
| **B:OR-14:8-438** | **FS-06-458** | Long bone fragment | L1 |  |  |
| **B:OR-14:8-439** | **FS-06-459** | Long bone fragment | L1 |  |  |
| **B:OR-14:8-440** | **FS-06-460** | tooth fragment | L1 |  |  |
| **B:OR-14:8-441** | **FS-06-461** | Long bone fragment | L1 |  |  |
| **B:OR-14:8-442** | **FS-06-462** | Long bone fragment | L1 |  |  |
| **B:OR-14:8-443** | **FS-06-463** | Cranial vault fragment | L1 |  |  |
| **B:OR-14:8-444** | **FS-06-464** | Rib Fragment | L1 |  |  |
| **B:OR-14:8-445** | **FS-06-465** | Long bone fragment | L1 |  |  |
| **B:OR-14:8-446** | **FS-06-466** | Long bone fragment | L1 |  |  |
| **B:OR-14:8-447** | **FS-06-467** | Rib Fragment | L1 |  |  |
| **B:OR-14:8-448** | **FS-06-468** | Long bone fragment | L1 |  |  |
| **B:OR-14:8-449** | **FS-06-469** | Cranial vault fragment | L1 |  |  |
| **B:OR-14:8-450** | **FS-06-470** | Long bone fragment | L1 |  |  |
| **B:OR-14:8-451** | **FS-06-471** | Long bone fragment | L1 |  |  |
| **B:OR-14:8-452** | **FS-06-472** | Long bone fragment | L1 |  |  |
| **B:OR-14:8-453** | **FS-06-473** | Cranial vault fragment | L1 |  |  |
| **B:OR-14:8-454** | **FS-06-474** | Long bone fragment | L1 |  |  |
| **B:OR-14:8-455** | **FS-06-475** | Long bone fragment | L1 |  |  |
| **B:OR-14:8-456** | **FS-06-476** | Rib Fragment | L1 |  |  |
| **B:OR-14:8-457** | **FS-06-477** | Long bone fragment | L1 |  |  |
| **B:OR-14:8-458** | **FS-06-478** | Rib Fragment | L1 |  |  |
| **B:OR-14:8-459** | **FS-06-479** | Cranial vault fragment | L1 |  |  |
| **B:OR-14:8-460** | **FS-06-480** | Vertebral Fragment | L1 |  |  |
| **B:OR-14:8-461** | **FS-06-481** | Vertebral Fragment | L1 |  |  |
| **B:OR-14:8-462** | **FS-06-482** | Bone Fragment | L1 |  |  |
| **B:OR-14:8-463** | **FS-06-483** | Long bone fragment | L1 |  |  |
| **B:OR-14:8-464** | **FS-06-484** | Long bone fragment | L1 |  |  |
| **B:OR-14:8-465** | **FS-06-485** | Rib Fragment | L1 |  |  |
| **B:OR-14:8-466** | **FS-06-486** | Bone Fragment | L1 |  |  |
| **B:OR-14:8-467** | **FS-06-487** | Long bone fragment | L1 |  |  |
| **B:OR-14:8-468** | **FS-06-488** | Cranial vault fragment | L1 |  |  |
| **B:OR-14:8-469** | **FS-06-489** | Non- identifiable | L1 |  |  |
| **B:OR-14:8-470** | **FS-06-490** | Rib Fragment | L1 |  |  |
| **B:OR-14:8-471** | **FS-06-491** | Non- identifiable | L1 |  |  |
| **B:OR-14:8-472** | **FS-06-492** | Non- identifiable | L1 |  |  |
| **B:OR-14:8-473** | **FS-06-493** | Non- identifiable | L1 |  |  |
| **B:OR-14:8-474** | **FS-06-494** | Non- identifiable | L1 |  |  |
| **B:OR-14:8-475** | **FS-06-495** | Non- identifiable | L1 |  |  |
| **B:OR-14:8-476** | **FS-06-496** | Non- identifiable | L1 |  |  |
| **B:OR-14:8-477** | **FS-06-497** | Non- identifiable | L1 |  |  |
| **B:OR-14:8-478** | **FS-06-498** | Non- identifiable | L1 |  |  |
| **B:OR-14:8-479** | **FS-06-499** | Non- identifiable | L1 |  |  |
| **B:OR-14:8-480** | **FS-06-500** | Non- identifiable | L1 |  |  |
| **B:OR-14:8-481** | **FS-06-501** | Non- identifiable | L1 |  |  |
| **B:OR-14:8-482** | **FS-06-502** | Non- identifiable | L1 |  |  |
| **B:OR-14:8-483** | **FS-06-503** | Non- identifiable | L1 |  |  |
| **B:OR-14:8-484** | **FS-06-504** | Non- identifiable | L1 |  |  |
| **B:OR-14:8-485** | **FS-06-505** | Tooth fragment | L1 |  |  |
| **B:OR-14:8-486** | **FS-06-506** | Non- identifiable | L1 |  |  |
| **B:OR-14:8-487** | **FS-06-507** | Non- identifiable | L1 |  |  |
| **B:OR-14:8-488** | **FS-06-508** | Non- identifiable | L1 |  |  |
| **B:OR-14:8-489** | **FS-06-509** | Non- identifiable | L1 |  |  |
| **B:OR-14:8-490** | **FS-06-510** | Non- identifiable | L1 |  |  |
| **B:OR-14:8-491** | **FS-06-511** | Non- identifiable | L1 |  |  |
| **B:OR-14:8-492** | **FS-06-512** | Non- identifiable | L1 |  |  |
| **B:OR-14:8-493** | **FS-06-513** | Non- identifiable | L1 |  |  |
| **B:OR-14:8-494** | **FS-06-514** | Non- identifiable | L1 |  |  |
| **B:OR-14:8-495** | **FS-06-515** | Non- identifiable | L1 |  |  |
| **B:OR-14:8-496** | **FS-06-516** | Tooth root fragment | L1 |  |  |
| **B:OR-14:8-497** | **FS-06-517** | Non- identifiable | L1 |  |  |
| **B:OR-14:8-498** | **FS-06-518** | Non- identifiable | L1 |  |  |
| **B:OR-14:8-499** | **FS-06-519** | Tooth root fragment | L1 |  |  |
| **B:OR-14:8-500** | **FS-06-520** | Rib Fragment | L1 |  |  |
| **B:OR-14:8-501** | **FS-06-521** | Right hamate | L1 |  |  |
| **B:OR-14:8-502** | **FS-06-522** | Metatarsal fragment | L1 |  |  |
| **B:OR-14:8-503** | **FS-06-523** | Metatarsal fragment | L1 |  |  |
| **B:OR-14:8-504** | **FS-06-524** | Long bone fragment | L2 | AMS Sample |  |
| **B:OR-14:8-505** | **FS-06-525** | Hand phalanx | L2 | AMS Sample |  |
| **B:OR-14:8-506** | **FS-06-526** | Metatarsal | L2 | AMS Sample |  |
| **B:OR-14:8-507** | **FS-06-527** | Rib Fragment | L2 | AMS Sample |  |
| **B:OR-14:8-508** | **FS-06-528** | Hand phalanx | L2 | AMS Sample |  |
| **B:OR-14:8-509** | **FS-06-529** | pedal 1st proximal phalanx | L3 | AMS Sample |  |
| **B:OR-14:8-510** | **FS-06-530** | Long bone fragment | L3 | AMS Sample |  |
| **B:OR-14:8-511** | **FS-06-531** | Rib Fragment | L3 | AMS Sample |  |
| **B:OR-14:8-512** | **FS-06-532** | Tarsal | L3 | AMS Sample |  |
| **B:OR-14:8-513** | **FS-06-533** | Long Bone Shaft | L3 | AMS Sample |  |
| **B:OR-14:8-514** | **FS-06-534** | LM^1^ | L3 | DNA |  |
| **B:OR-14:8-515** | **FS-06-535** | RP (max) | L3 | DNA |  |
| **B:OR-14:8-516** | **FS-06-536** | LC (max) | L3 | DNA |  |
| **B:OR-14:8-517** | **FS-06-537** | LI^1^ | L3 | DNA |  |
| **B:OR-14:8-518** | **FS-06-538** | LC_1_ | L3 | DNA |  |
| **B:OR-14:8-519** | **FS-06-539** | Humeral shaft | L4 | AMS Sample |  |
| **B:OR-14:8-520** | **FS-06-540** | Cranial vault fragment | L4 | AMS Sample |  |
| **B:OR-14:8-521** | **FS-06-541** | Rib Fragment | L4 | AMS Sample |  |
| **B:OR-14:8-522** | **FS-06-542** | R 3rd metacarpal | L4 | AMS Sample |  |
| **B:OR-14:8-523** | **FS-06-543** | Rib Fragment | L4 | AMS Sample |  |
| **B:OR-14:8-524** | **FS-06-544** | Rm^2^ | L4 | DNA |  |
| **B:OR-14:8-525** | **FS-06-545** | LM^2/3^ | L4 | DNA |  |
| **B:OR-14:8-526** | **FS-06-546** | RM^2/3^ | L4 | DNA |  |
| **B:OR-14:8-527** | **FS-06-547** | LI1 | L4 | DNA |  |
| **B:OR-14:8-528** | **FS-06-548** | RM^1^ | L4 | DNA |  |
| **B:OR-14:8-529** | **FS-06-549** | Long bone fragment | L5 | AMS Sample |  |
| **B:OR-14:8-530** | **FS-06-550** | Ulna shaft | L5 | AMS Sample |  |
| **B:OR-14:8-531** | **FS-06-551** | 4th Metacarpal | L5 | AMS Sample |  |
| **B:OR-14:8-532** | **FS-06-552** | Rib Fragment | L5 | AMS Sample |  |
| **B:OR-14:8-533** | **FS-06-553** | Rib Fragment | L5 | AMS Sample |  |
| **B:OR-14:8-534** | **FS-06-554** | RI^1^ | L5 | DNA |  |
| **B:OR-14:8-535** | **FS-06-555** | RM^2^ | L5 | DNA |  |
| **B:OR-14:8-536** | **FS-06-556** | Long bone fragment | L2 |  |  |
| **B:OR-14:8-537** | **FS-06-557** | RM^2^ | L2 |  | **Y** |
| **B:OR-14:8-538** | **FS-06-558** | RP_3_ | L2 |  | **Y** |
| **B:OR-14:8-539** | **FS-06-559** | RI^2^ | L2 |  | **Y** |
| **B:OR-14:8-540** | **FS-06-560** | Cranial vault fragment | L2 |  |  |
| **B:OR-14:8-541** | **FS-06-561** | Long bone fragment | L2 |  |  |
| **B:OR-14:8-542** | **FS-06-562** | Pedal Phalanx | L2 |  |  |
| **B:OR-14:8-543** | **FS-06-563** | LP_4_ | L2 |  | **Y** |
| **B:OR-14:8-544** | **FS-06-564** | Rib Fragment | L2 |  |  |
| **B:OR-14:8-545** | **FS-06-565** | Cranial vault fragment | L2 |  |  |
| **B:OR-14:8-546** | **FS-06-566** | Rib Fragment | L2 |  |  |
| **B:OR-14:8-547** | **FS-06-567** | Cranial vault fragment | L2 |  |  |
| **B:OR-14:8-548** | **FS-06-568** | phalanx | L2 |  |  |
| **B:OR-14:8-549** | **FS-06-569** | Long bone fragment | L2 |  |  |
| **B:OR-14:8-550** | **FS-06-570** | Cranial vault fragment | L2 |  |  |
| **B:OR-14:8-551** | **FS-06-571** | phalanx | L2 |  |  |
| **B:OR-14:8-552** | **FS-06-572** | Rib Fragment | L2 |  |  |
| **B:OR-14:8-553** | **FS-06-573** | Long bone fragment | L2 |  |  |
| **B:OR-14:8-554** | **FS-06-574** | Rib Fragment | L2 | Juvenile |  |
| **B:OR-14:8-555** | **FS-06-575** | Rib Fragment | L2 |  |  |
| **B:OR-14:8-556** | **FS-06-576** | Rib Fragment | L2 |  |  |
| **B:OR-14:8-557** | **FS-06-577** | Long bone fragment | L2 |  |  |
| **B:OR-14:8-558** | **FS-06-578** | Rib Fragment | L2 |  |  |
| **B:OR-14:8-559** | **FS-06-579** | Cranial vault fragment | L2 |  |  |
| **B:OR-14:8-560** | **FS-06-580** | Left Clavicle shaft | L2 |  |  |
| **B:OR-14:8-561** | **FS-06-581** | Distal Pedal Phalanx | L2 |  |  |
| **B:OR-14:8-562** | **FS-06-582** | Right Distal radius | L2 | 3 pieces |  |
| **B:OR-14:8-563** | **FS-06-583** | Right Metatarsal | L2 |  |  |
| **B:OR-14:8-564** | **FS-06-584** | Navicular | L2 |  |  |
| **B:OR-14:8-565** | **FS-06-585** | Femoral shaft fragment | L2 |  |  |
| **B:OR-14:8-566** | **FS-06-586** | Navicular | L2 |  |  |
| **B:OR-14:8-567** | **FS-06-587** | manual 1st distal Phalanx | L2 |  |  |
| **B:OR-14:8-568** | **FS-06-588** | Right scaphoid | L2 |  |  |
| **B:OR-14:8-569** | **FS-06-589** | Right scaphoid | L2 |  |  |
| **B:OR-14:8-570** | **FS-06-590** | Vertebral Fragment | L2 |  |  |
| **B:OR-14:8-571** | **FS-06-591** | Vertebral Fragment | L2 | Juvenile |  |
| **B:OR-14:8-572** | **FS-06-592** | metacarpal | L2 |  |  |
| **B:OR-14:8-573** | **FS-06-593** | Rib Fragment | L2 | Juvenile |  |
| **B:OR-14:8-574** | **FS-06-594** | Bone Fragment | Below Skull level - from Cave extension |  |  |
| **B:OR-14:8-575** | **FS-06-595** | Bone Fragment | Below Skull level - from Cave extension |  |  |
| **B:OR-14:8-576** | **FS-06-596** | Bone Fragment | Below Skull level - from Cave extension |  |  |
| **B:OR-14:8-577** | **FS-06-597** | Bone Fragment | Below Skull level - from Cave extension |  |  |
| **B:OR-14:8-578** | **FS-06-598** | Bone Fragment | Below Skull level - from Cave extension |  |  |
| **B:OR-14:8-579** | **FS-06-599** | Bone Fragment | Below Skull level - from Cave extension |  |  |
| **B:OR-14:8-580** | **FS-06-600** | Bone Fragment | Below Skull level - from Cave extension |  |  |
| **B:OR-14:8-581** | **FS-06-601** | Bone Fragment | Below Skull level - from Cave extension |  |  |
| **B:OR-14:8-582** | **FS-06-602** | Bone Fragment | Below Skull level - from Cave extension |  |  |
| **B:OR-14:8-583** | **FS-06-603** | Bone Fragment | Below Skull level - from Cave extension |  |  |
| **B:OR-14:8-584** | **FS-06-604** | Bone Fragment | Below Skull level - from Cave extension |  |  |
| **B:OR-14:8-585** | **FS-06-605** | Hand phalanx | LEVEL 2 |  |  |
| **B:OR-14:8-586** | **FS-06-606** | Cranial vault fragment | L2 |  |  |
| **B:OR-14:8-587** | **FS-06-607** | LM_1/2_ | L2 |  | **Y** |
| **B:OR-14:8-588** | **FS-06-608** | Vertebral Fragment | L2 |  |  |
| **B:OR-14:8-589** | **FS-06-609** | metacarpal | L2 |  |  |
| **B:OR-14:8-590** | **FS-06-610** | Hand phalanx | L2 |  |  |
| **B:OR-14:8-591** | **FS-06-611** | Left scaphoid | L2 |  |  |
| **B:OR-14:8-592** | **FS-06-612** | Long bone fragment | L2 |  |  |
| **B:OR-14:8-593** | **FS-06-613** | Long bone fragment | L2 |  |  |
| **B:OR-14:8-594** | **FS-06-614** | Rib Fragment | L2 |  |  |
| **B:OR-14:8-595** | **FS-06-615** | Long bone fragment | L2 |  |  |
| **B:OR-14:8-596** | **FS-06-616** | Tibial Shaft | From Skull block" Cave extension" |  |  |
| **B:OR-14:8-597** | **FS-06-617** | Long bone fragment | L2 |  |  |
| **B:OR-14:8-598** | **FS-06-618** | metatarsal head | L2 |  |  |
| **B:OR-14:8-599** | **FS-06-619** | Hand phalanx | L2 |  |  |
| **B:OR-14:8-600** | **FS-06-620** | Hand phalanx | L2 |  |  |
| **B:OR-14:8-601** | **FS-06-621** | Hand phalanx | L2 |  |  |
| **B:OR-14:8-602** | **FS-06-622** | Hand phalanx | L2 |  |  |
| **B:OR-14:8-603** | **FS-06-623** | Hand phalanx | L2 |  |  |
| **B:OR-14:8-604** | **FS-06-624** | Hand phalanx | L2 |  |  |
| **B:OR-14:8-605** | **FS-06-625** | Vertebral Fragment | L2 |  |  |
| **B:OR-14:8-606** | **FS-06-626** | Vertebral Fragment | L2 | c2 |  |
| **B:OR-14:8-607** | **FS-06-627** | Bone Fragment | L2 |  |  |
| **B:OR-14:8-608** | **FS-06-628** | Long bone fragment | L2 |  |  |
| **B:OR-14:8-609** | **FS-06-629** | Long bone fragment | L2 |  |  |
| **B:OR-14:8-610** | **FS-06-630** | Vertebral Fragment | L2 | Juvenile |  |
| **B:OR-14:8-611** | **FS-06-631** | Vertebral Fragment | L2 |  |  |
| **B:OR-14:8-612** | **FS-06-632** | Vertebral Fragment | L2 |  |  |
| **B:OR-14:8-613** | **FS-06-633** | pedal Phalanx | L2 |  |  |
| **B:OR-14:8-614** | **FS-06-634** | distal hand phalanx | L2 |  |  |
| **B:OR-14:8-615** | **FS-06-635** | Long bone fragment | L2 |  |  |
| **B:OR-14:8-616** | **FS-06-636** | Bone Fragment | L2 |  |  |
| **B:OR-14:8-617** | **FS-06-637** | Long bone fragment | L2 |  |  |
| **B:OR-14:8-618** | **FS-06-638** | Long bone fragment | L2 |  |  |
| **B:OR-14:8-619** | **FS-06-639** | Long bone fragment | L2 |  |  |
| **B:OR-14:8-620** | **FS-06-640** | Long bone fragment | L2 |  |  |
| **B:OR-14:8-621** | **FS-06-641** | Long bone fragment | L2 |  |  |
| **B:OR-14:8-622** | **FS-06-642** | Long bone fragment | L2 |  |  |
| **B:OR-14:8-623** | **FS-06-643** | Long bone fragment | L2 |  |  |
| **B:OR-14:8-624** | **FS-06-644** | Bone Fragment | L2 |  |  |
| **B:OR-14:8-625** | **FS-06-645** | Long bone fragment | L2 |  |  |
| **B:OR-14:8-626** | **FS-06-646** | Cranial vault fragment | L2 |  |  |
| **B:OR-14:8-627** | **FS-06-647** | Long bone fragment | L2 |  |  |
| **B:OR-14:8-628** | **FS-06-648** | Bone Fragment | L2 |  |  |
| **B:OR-14:8-629** | **FS-06-649** | Distal Pedal phalanx | L2 |  |  |
| **B:OR-14:8-630** | **FS-06-650** | Hand phalanx | L2 |  |  |
| **B:OR-14:8-631** | **FS-06-651** | Cranial vault fragment | L2 |  |  |
| **B:OR-14:8-632** | **FS-06-652** | Vertebral Fragment | L2 | juvenile |  |
| **B:OR-14:8-633** | **FS-06-653** | Long bone fragment | L2 |  |  |
| **B:OR-14:8-634** | **FS-06-654** | Bone Fragment | L2 |  |  |
| **B:OR-14:8-635** | **FS-06-655** | Bone Fragment | L2 |  |  |
| **B:OR-14:8-636** | **FS-06-656** | Cranial vault fragment | L2 |  |  |
| **B:OR-14:8-637** | **FS-06-657** | Non- identifiable | L2 |  |  |
| **B:OR-14:8-638** | **FS-06-658** | Bone Fragment | L2 |  |  |
| **B:OR-14:8-639** | **FS-06-659** | Long bone fragment | L2 |  |  |
| **B:OR-14:8-640** | **FS-06-660** | Long bone fragment | L2 |  |  |
| **B:OR-14:8-641** | **FS-06-661** | Cranial vault fragment | L2 |  |  |
| **B:OR-14:8-642** | **FS-06-662** | Cranial vault fragment | L2 |  |  |
| **B:OR-14:8-643** | **FS-06-663** | Cranial vault fragment | L2 |  |  |
| **B:OR-14:8-644** | **FS-06-664** | Cranial vault fragment | L6 |  |  |
| **B:OR-14:8-645** | **FS-06-665** | Long bone fragment | L2 |  |  |
| **B:OR-14:8-646** | **FS-06-666** | Cranial vault fragment | L2 |  |  |
| **B:OR-14:8-647** | **FS-06-667** | Distal Pedal phalanx | L2 |  |  |
| **B:OR-14:8-648** | **FS-06-668** | Rib Fragment | L2 |  |  |
| **B:OR-14:8-649** | **FS-06-669** | Long bone fragment | L2 |  |  |
| **B:OR-14:8-650** | **FS-06-670** | Non- identifiable | L2 |  |  |
| **B:OR-14:8-651** | **FS-06-671** | Long bone fragment | L2 |  |  |
| **B:OR-14:8-652** | **FS-06-672** | Long bone fragment | L2 |  |  |
| **B:OR-14:8-653** | **FS-06-673** | Long bone fragment | L2 |  |  |
| **B:OR-14:8-654** | **FS-06-674** | Long bone fragment | L2 |  |  |
| **B:OR-14:8-655** | **FS-06-675** | Long bone fragment | L2 |  |  |
| **B:OR-14:8-656** | **FS-06-676** | Long bone fragment | L2 |  |  |
| **B:OR-14:8-657** | **FS-06-677** | Non- identifiable | L2 |  |  |
| **B:OR-14:8-658** | **FS-06-678** | Cranial vault fragment | L2 | juvenile |  |
| **B:OR-14:8-659** | **FS-06-679** | Non- identifiable | L2 |  |  |
| **B:OR-14:8-660** | **FS-06-680** | Long bone fragment | L2 |  |  |
| **B:OR-14:8-661** | **FS-06-681** | Distal Hand phalanx | L2 |  |  |
| **B:OR-14:8-662** | **FS-06-682** | Long bone fragment | L2 |  |  |
| **B:OR-14:8-663** | **FS-06-683** | Long bone fragment | L2 |  |  |
| **B:OR-14:8-664** | **FS-06-684** | Long bone fragment | L2 |  |  |
| **B:OR-14:8-665** | **FS-06-685** | Long bone fragment | L2 |  |  |
| **B:OR-14:8-666** | **FS-06-686** | Long bone fragment | L2 |  |  |
| **B:OR-14:8-667** | **FS-06-687** | Long bone fragment | L2 |  |  |
| **B:OR-14:8-668** | **FS-06-688** | Cranial vault fragment | L2 |  |  |
| **B:OR-14:8-669** | **FS-06-689** | Long bone fragment | L2 |  |  |
| **B:OR-14:8-670** | **FS-06-690** | Cranial vault fragment | L2 |  |  |
| **B:OR-14:8-671** | **FS-06-691** | Long bone fragment | L2 |  |  |
| **B:OR-14:8-672** | **FS-06-692** | Cranial vault fragment | L2 |  |  |
| **B:OR-14:8-673** | **FS-06-693** | Long bone fragment | L2 |  |  |
| **B:OR-14:8-674** | **FS-06-694** | Long bone fragment | L2 |  |  |
| **B:OR-14:8-675** | **FS-06-695** | Long bone fragment | L2 |  |  |
| **B:OR-14:8-676** | **FS-06-696** | Fish Bone | L2 |  |  |
| **B:OR-14:8-677** | **FS-06-697** | Long bone fragment | L2 |  |  |
| **B:OR-14:8-678** | **FS-06-698** | Non- identifiable | L2 |  |  |
| **B:OR-14:8-679** | **FS-06-699** | Long bone fragment | L2 |  |  |
| **B:OR-14:8-680** | **FS-06-700** | Non- identifiable | L2 |  |  |
| **B:OR-14:8-681** | **FS-06-701** | Cranial vault fragment | L2 |  |  |
| **B:OR-14:8-682** | **FS-06-702** | Non- identifiable | L2 |  |  |
| **B:OR-14:8-683** | **FS-06-703** | Non- identifiable | L2 |  |  |
| **B:OR-14:8-684** | **FS-06-704** | Long bone fragment | L2 |  |  |
| **B:OR-14:8-685** | **FS-06-705** | Long bone fragment | L2 |  |  |
| **B:OR-14:8-686** | **FS-06-706** | Non- identifiable | L2 |  |  |
| **B:OR-14:8-687** | **FS-06-707** | Non- identifiable | L2 |  |  |
| **B:OR-14:8-688** | **FS-06-708** | Non- identifiable | L2 |  |  |
| **B:OR-14:8-689** | **FS-06-709** | Non- identifiable | L2 |  |  |
| **B:OR-14:8-690** | **FS-06-710** | Long bone fragment | L2 |  |  |
| **B:OR-14:8-691** | **FS-06-711** | Non- identifiable | L2 |  |  |
| **B:OR-14:8-692** | **FS-06-712** | Non- identifiable | L2 |  |  |
| **B:OR-14:8-693** | **FS-06-713** | Non- identifiable | L2 |  |  |
| **B:OR-14:8-694** | **FS-06-714** | Non- identifiable | L2 |  |  |
| **B:OR-14:8-695** | **FS-06-715** | Left proximal Ulna | L3 |  |  |
| **B:OR-14:8-696** | **FS-06-716** | Clavicle | L3 |  |  |
| **B:OR-14:8-697** | **FS-06-717** | Distal Humerus | L3 | Right |  |
| **B:OR-14:8-698** | **FS-06-718** | Hand phalanx | L3 |  |  |
| **B:OR-14:8-699** | **FS-06-719** | Hand phalanx | L3 |  |  |
| **B:OR-14:8-700** | **FS-06-720** | L 3rd Metacarpal | L3 |  |  |
| **B:OR-14:8-701** | **FS-06-721** | Left Humeral shaft | L3 |  |  |
| **B:OR-14:8-702** | **FS-06-722** | Metatarsal | L3 |  |  |
| **B:OR-14:8-703** | **FS-06-723** | L Radius | L3 | juvenile |  |
| **B:OR-14:8-704** | **FS-06-724** | Long bone fragment | L3 |  |  |
| **B:OR-14:8-705** | **FS-06-725** | R proximal Femur | L3 | juvenile |  |
| **B:OR-14:8-706** | **FS-06-726** | Pedal Phalanx | L3 |  |  |
| **B:OR-14:8-707** | **FS-06-727** | Hand phalanx | L3 |  |  |
| **B:OR-14:8-708** | **FS-06-728** | Hand phalanx | L3 |  |  |
| **B:OR-14:8-709** | **FS-06-729** | Hand phalanx | L3 |  |  |
| **B:OR-14:8-710** | **FS-06-730** | Rib Fragment | L3 |  |  |
| **B:OR-14:8-711** | **FS-06-731** | Metatarsal head | L3 |  |  |
| **B:OR-14:8-712** | **FS-06-732** | Rib Fragment | L3 |  |  |
| **B:OR-14:8-713** | **FS-06-733** | Os coxa | L3 | juvenile |  |
| **B:OR-14:8-714** | **FS-06-734** | Non- identifiable | L3 |  |  |
| **B:OR-14:8-715** | **FS-06-735** | Pedal Phalanx | L3 |  |  |
| **B:OR-14:8-716** | **FS-06-736** | L Capitate | L3 |  | **Y** |
| **B:OR-14:8-717** | **FS-06-737** | Long bone fragment | L3 | juvenile |  |
| **B:OR-14:8-718** | **FS-06-738** | Cranial vault fragment | L3 |  |  |
| **B:OR-14:8-719** | **FS-06-739** | Long bone fragment | L3 |  |  |
| **B:OR-14:8-720** | **FS-06-740** | Rib Fragment | L3 |  |  |
| **B:OR-14:8-721** | **FS-06-741** | L Lunate | L3 |  | **Y** |
| **B:OR-14:8-722** | **FS-06-742** | L Hamate | L3 |  | **Y** |
| **B:OR-14:8-723** | **FS-06-743** | Os coxa | L3 | juvenile |  |
| **B:OR-14:8-724** | **FS-06-744** | Hand phalanx | L3 |  |  |
| **B:OR-14:8-725** | **FS-06-745** | Long bone fragment | L3 |  |  |
| **B:OR-14:8-726** | **FS-06-746** | Metacarpal head | L3 |  |  |
| **B:OR-14:8-727** | **FS-06-747** | Hand phalanx | L3 | 4 pieces |  |
| **B:OR-14:8-728** | **FS-06-748** | Hand phalanx | L3 |  |  |
| **B:OR-14:8-729** | **FS-06-749** | Vertebral Fragment | L3 | juvenile |  |
| **B:OR-14:8-730** | **FS-06-750** | L Triquetra | L3 |  | **Y** |
| **B:OR-14:8-732** | **FS-06-752** | Non- identifiable | L3 |  |  |
| **B:OR-14:8-733** | **FS-06-753** | Os coxa | L3 | juvenile |  |
| **B:OR-14:8-734** | **FS-06-754** | Hand phalanx | L3 |  |  |
| **B:OR-14:8-735** | **FS-06-755** | 4th Metacarpal | L3 |  | **Y** |
| **B:OR-14:8-736** | **FS-06-756** | Hand phalanx | L3 |  |  |
| **B:OR-14:8-737** | **FS-06-757** | 4th Metacarpal | L3 |  | **Y** |
| **B:OR-14:8-738** | **FS-06-758** | Hand phalanx | L3 |  |  |
| **B:OR-14:8-739** | **FS-06-759** | Long bone fragment | L3 |  |  |
| **B:OR-14:8-740** | **FS-06-760** | Distal hand phalanx | L3 |  |  |
| **B:OR-14:8-741** | **FS-06-761** | Rib Fragment | L3 |  |  |
| **B:OR-14:8-742** | **FS-06-762** | Metacarpal | L3 |  |  |
| **B:OR-14:8-743** | **FS-06-763** | Rib Fragment | L3 | juvenile |  |
| **B:OR-14:8-744** | **FS-06-764** | Carpal | L3 | juvenile |  |
| **B:OR-14:8-745** | **FS-06-765** | Cranial vault fragment | L3 |  |  |
| **B:OR-14:8-746** | **FS-06-766** | Distal Hand phalanx | L3 |  |  |
| **B:OR-14:8-747** | **FS-06-767** | Vertebral Fragment | L3 | juvenile |  |
| **B:OR-14:8-748** | **FS-06-768** | Metatarsal | L3 | juvenile |  |
| **B:OR-14:8-749** | **FS-06-769** | Rib Fragment | L3 |  |  |
| **B:OR-14:8-750** | **FS-06-770** | Left scaphoid | L3 |  |  |
| **B:OR-14:8-751** | **FS-06-771** | Interm. Hand phalanx | L3 |  |  |
| **B:OR-14:8-752** | **FS-06-772** | Long bone fragment | L3 |  |  |
| **B:OR-14:8-753** | **FS-06-773** | Long bone fragment | L3 |  |  |
| **B:OR-14:8-754** | **FS-06-774** | Cranial vault fragment | L3 |  |  |
| **B:OR-14:8-755** | **FS-06-775** | Rib Fragment | L3 | juvenile |  |
| **B:OR-14:8-756** | **FS-06-776** | Long bone fragment | L3 |  |  |
| **B:OR-14:8-757** | **FS-06-777** | Metacarpal head | L3 |  |  |
| **B:OR-14:8-758** | **FS-06-778** | Long bone fragment | L3 |  |  |
| **B:OR-14:8-759** | **FS-06-779** | Long bone fragment | L3 | juvenile |  |
| **B:OR-14:8-760** | **FS-06-780** | Non- identifiable | L3 |  |  |
| **B:OR-14:8-761** | **FS-06-781** | Long bone fragment | L3 | juvenile |  |
| **B:OR-14:8-762** | **FS-06-782** | Cranial vault fragment | L3 |  |  |
| **B:OR-14:8-763** | **FS-06-783** | Long bone fragment | L3 | juvenile |  |
| **B:OR-14:8-764** | **FS-06-784** | Distal hand phalanx | L3 |  |  |
| **B:OR-14:8-765** | **FS-06-785** | Vertebral Fragment | L3 | juvenile |  |
| **B:OR-14:8-766** | **FS-06-786** | Rib Fragment | L3 |  |  |
| **B:OR-14:8-767** | **FS-06-787** | Cranial vault fragment | L3 |  |  |
| **B:OR-14:8-768** | **FS-06-788** | Long bone fragment | L3 |  |  |
| **B:OR-14:8-769** | **FS-06-789** | Vertebral Fragment | L3 | juvenile |  |
| **B:OR-14:8-770** | **FS-06-790** | Rib Fragment | L3 | juvenile |  |
| **B:OR-14:8-771** | **FS-06-791** | Mandible right | L4 | Mandible contains the following teeth: RI_1/2_, RC_1_, RP_3/4_, RM_1/2_ Measurements taken on all teeth | **Y** |
| **B:OR-14:8-772** | **FS-06-792** | Mandible | Skull block "Cave extension" |  |  |
| **B:OR-14:8-773** | **FS-06-793** | Vertebral Fragment | L3 | juvenile |  |
| **B:OR-14:8-774** | **FS-06-794** | Pedal Phalanx | L3 |  |  |
| **B:OR-14:8-775** | **FS-06-795** | Left Trapezium | L3 |  | **Y** |
| **B:OR-14:8-776** | **FS-06-796** | Rib Fragment | L3 |  |  |
| **B:OR-14:8-777** | **FS-06-797** | Vertebral Fragment | L3 | juvenile |  |
| **B:OR-14:8-778** | **FS-06-798** | Right Triquetral | L3 |  | **Y** |
| **B:OR-14:8-779** | **FS-06-799** | Carpal | L3 |  |  |
| **B:OR-14:8-780** | **FS-06-800** | Interm. Hand phalanx | L3 | juvenile |  |
| **B:OR-14:8-781** | **FS-06-801** | proximal Hand phalanx | L3 | juvenile |  |
| **B:OR-14:8-782** | **FS-06-802** | Rib Fragment | L3 | juvenile |  |
| **B:OR-14:8-783** | **FS-06-803** | Rib Fragment | L3 | juvenile |  |
| **B:OR-14:8-784** | **FS-06-804** | Vertebral Fragment | L3 |  |  |
| **B:OR-14:8-785** | **FS-06-805** | Rib Fragment | L3 |  |  |
| **B:OR-14:8-786** | **FS-06-806** | Vertebral Fragment | L3 |  |  |
| **B:OR-14:8-787** | **FS-06-807** | Vertebral Fragment | L3 |  |  |
| **B:OR-14:8-788** | **FS-06-808** | Long bone fragment | L3 |  |  |
| **B:OR-14:8-789** | **FS-06-809** | Interm. Hand phalanx | L3 |  |  |
| **B:OR-14:8-790** | **FS-06-810** | Rib Fragment | L3 | juvenile |  |
| **B:OR-14:8-791** | **FS-06-811** | Sternabra | L3 | juvenile |  |
| **B:OR-14:8-792** | **FS-06-812** | Long bone fragment | L3 |  |  |
| **B:OR-14:8-793** | **FS-06-813** | Long bone fragment | L3 |  |  |
| **B:OR-14:8-794** | **FS-06-814** | Long bone fragment | L3 |  |  |
| **B:OR-14:8-795** | **FS-06-815** | Long bone fragment | L3 |  |  |
| **B:OR-14:8-796** | **FS-06-816** | Rib Fragment | L3 |  |  |
| **B:OR-14:8-797** | **FS-06-817** | Cranial vault fragment | L3 |  |  |
| **B:OR-14:8-798** | **FS-06-818** | Rib Fragment | L3 |  |  |
| **B:OR-14:8-799** | **FS-06-819** | Long bone fragment | L3 |  |  |
| **B:OR-14:8-800** | **FS-06-820** | Interm. Hand phalanx | L3 |  |  |
| **B:OR-14:8-801** | **FS-06-821** | Interm. Hand phalanx | L3 |  |  |
| **B:OR-14:8-802** | **FS-06-822** | Cranial vault fragment | L3 |  |  |
| **B:OR-14:8-803** | **FS-06-823** | Metacarpal | L3 |  |  |
| **B:OR-14:8-804** | **FS-06-824** | Long bone fragment | L3 |  |  |
| **B:OR-14:8-805** | **FS-06-825** | Long bone fragment | L3 |  |  |
| **B:OR-14:8-806** | **FS-06-826** | Rib Fragment | L3 |  |  |
| **B:OR-14:8-807** | **FS-06-827** | Rib Fragment | L3 |  |  |
| **B:OR-14:8-808** | **FS-06-828** | Rib Fragment | L3 |  |  |
| **B:OR-14:8-809** | **FS-06-829** | Vertebral Fragment | L3 |  |  |
| **B:OR-14:8-810** | **FS-06-830** | Rib Fragment | L3 |  |  |
| **B:OR-14:8-811** | **FS-06-831** | Long bone fragment | L3 |  |  |
| **B:OR-14:8-812** | **FS-06-832** | Cranial vault fragment | L3 |  |  |
| **B:OR-14:8-813** | **FS-06-833** | Long bone fragment | L3 |  |  |
| **B:OR-14:8-814** | **FS-06-834** | Long bone fragment | L3 |  |  |
| **B:OR-14:8-815** | **FS-06-835** | Long bone fragment | L3 |  |  |
| **B:OR-14:8-816** | **FS-06-836** | Long bone fragment | L3 |  |  |
| **B:OR-14:8-817** | **FS-06-837** | Long bone fragment | L3 |  |  |
| **B:OR-14:8-818** | **FS-06-838** | Long bone fragment | L3 |  |  |
| **B:OR-14:8-819** | **FS-06-839** | Rib Fragment | L3 |  |  |
| **B:OR-14:8-820** | **FS-06-840** | Rib Fragment | L3 |  |  |
| **B:OR-14:8-821** | **FS-06-841** | Long bone fragment | L3 |  |  |
| **B:OR-14:8-822** | **FS-06-842** | Long bone fragment | L3 |  |  |
| **B:OR-14:8-823** | **FS-06-843** | Vertebral Fragment | L3 |  |  |
| **B:OR-14:8-824** | **FS-06-844** | Rib Fragment | L3 |  |  |
| **B:OR-14:8-825** | **FS-06-845** | Long bone fragment | L3 |  |  |
| **B:OR-14:8-826** | **FS-06-846** | Non- identifiable | L3 |  |  |
| **B:OR-14:8-827** | **FS-06-847** | Non- identifiable | L3 |  |  |
| **B:OR-14:8-828** | **FS-06-848** | Non- identifiable | L3 |  |  |
| **B:OR-14:8-829** | **FS-06-849** | Non- identifiable | L3 |  |  |
| **B:OR-14:8-830** | **FS-06-850** | Non- identifiable | L3 |  |  |
| **B:OR-14:8-831** | **FS-06-851** | Non- identifiable | L3 |  |  |
| **B:OR-14:8-832** | **FS-06-852** | Non- identifiable | L3 |  |  |
| **B:OR-14:8-833** | **FS-06-853** | Non- identifiable | L3 |  |  |
| **B:OR-14:8-834** | **FS-06-854** | Non- identifiable | L3 |  |  |
| **B:OR-14:8-835** | **FS-06-855** | Non- identifiable | L3 |  |  |
| **B:OR-14:8-836** | **FS-06-856** | Non- identifiable | L3 |  |  |
| **B:OR-14:8-837** | **FS-06-857** | Non- identifiable | L3 |  |  |
| **B:OR-14:8-838** | **FS-06-858** | Non- identifiable | L3 |  |  |
| **B:OR-14:8-839** | **FS-06-859** | Non- identifiable | L3 |  |  |
| **B:OR-14:8-840** | **FS-06-860** | Non- identifiable | L3 |  |  |
| **B:OR-14:8-841** | **FS-06-861** | Non- identifiable | L3 |  |  |
| **B:OR-14:8-842** | **FS-06-862** | Long bone fragment | L3 |  |  |
| **B:OR-14:8-843** | **FS-06-863** | Long bone fragment | L3 |  |  |
| **B:OR-14:8-844** | **FS-06-864** | Long bone fragment | L3 |  |  |
| **B:OR-14:8-845** | **FS-06-865** | Long bone fragment | L3 |  |  |
| **B:OR-14:8-846** | **FS-06-866** | Long bone fragment | L3 |  |  |
| **B:OR-14:8-847** | **FS-06-867** | Long bone fragment | L3 |  |  |
| **B:OR-14:8-848** | **FS-06-868** | Long bone fragment | L3 |  |  |
| **B:OR-14:8-849** | **FS-06-869** | Long bone fragment | L3 |  |  |
| **B:OR-14:8-850** | **FS-06-870** | Long bone fragment | L3 |  |  |
| **B:OR-14:8-851** | **FS-06-871** | Long bone fragment | L3 |  |  |
| **B:OR-14:8-852** | **FS-06-872** | Long bone fragment | L3 |  |  |
| **B:OR-14:8-853** | **FS-06-873** | Long bone fragment | L3 |  |  |
| **B:OR-14:8-854** | **FS-06-874** | Rib Fragment | L3 |  |  |
| **B:OR-14:8-855** | **FS-06-875** | Rib Fragment | L3 |  |  |
| **B:OR-14:8-856** | **FS-06-876** | Rib Fragment | L3 |  |  |
| **B:OR-14:8-857** | **FS-06-877** | Rib Fragment | L3 |  |  |
| **B:OR-14:8-858** | **FS-06-878** | Non- identifiable | L3 |  |  |
| **B:OR-14:8-859** | **FS-06-879** | Non- identifiable | L3 |  |  |
| **B:OR-14:8-860** | **FS-06-880** | Non- identifiable | L3 |  |  |
| **B:OR-14:8-861** | **FS-06-881** | Non- identifiable | L3 |  |  |
| **B:OR-14:8-862** | **FS-06-882** | Non- identifiable | L3 |  |  |
| **B:OR-14:8-863** | **FS-06-883** | Non- identifiable | L3 |  |  |
| **B:OR-14:8-864** | **FS-06-884** | Non- identifiable | L3 |  |  |
| **B:OR-14:8-865** | **FS-06-885** | Non- identifiable | L3 |  |  |
| **B:OR-14:8-866** | **FS-06-886** | Non- identifiable | L3 |  |  |
| **B:OR-14:8-867** | **FS-06-887** | Non- identifiable | L3 |  |  |
| **B:OR-14:8-868** | **FS-06-888** | Non- identifiable | L3 |  |  |
| **B:OR-14:8-869** | **FS-06-889** | Non- identifiable | L3 |  |  |
| **B:OR-14:8-870** | **FS-06-890** | Non- identifiable | L3 |  |  |
| **B:OR-14:8-871** | **FS-06-891** | Non- identifiable | L3 |  |  |
| **B:OR-14:8-872** | **FS-06-892** | Non- identifiable | L3 |  |  |
| **B:OR-14:8-873** | **FS-06-893** | Non- identifiable | L3 |  |  |
| **B:OR-14:8-874** | **FS-06-894** | Non- identifiable | L3 |  |  |
| **B:OR-14:8-875** | **FS-06-895** | Non- identifiable | L3 |  |  |
| **B:OR-14:8-876** | **FS-06-896** | Non- identifiable | L3 |  |  |
| **B:OR-14:8-877** | **FS-06-897** | Non- identifiable | L3 |  |  |
| **B:OR-14:8-878** | **FS-06-898** | Non- identifiable | L3 |  |  |
| **B:OR-14:8-879** | **FS-06-899** | Non- identifiable | L3 |  |  |
| **B:OR-14:8-880** | **FS-06-900** | Non- identifiable | L3 |  |  |
| **B:OR-14:8-881** | **FS-06-901** | Non- identifiable | L3 |  |  |
| **B:OR-14:8-882** | **FS-06-902** | Non- identifiable | L3 |  |  |
| **B:OR-14:8-883** | **FS-06-903** | Non- identifiable | L3 |  |  |
| **B:OR-14:8-884** | **FS-06-904** | Non- identifiable | L3 |  |  |
| **B:OR-14:8-885** | **FS-06-905** | Non- identifiable | L3 |  |  |
| **B:OR-14:8-886** | **FS-06-906** | Non- identifiable | L3 |  |  |
| **B:OR-14:8-887** | **FS-06-907** | Non- identifiable | L3 |  |  |
| **B:OR-14:8-888** | **FS-06-908** | RP_3_ | L3 |  | **Y** |
| **B:OR-14:8-889** | **FS-06-909** | RI_1_ | L3 |  | **Y** |
| **B:OR-14:8-890** | **FS-06-910** | RC_1_ | L3 |  |  |
| **B:OR-14:8-891** | **FS-06-911** | Right Distal humerus | L4 |  | **Y** |
| **B:OR-14:8-892** | **FS-06-912** | Right Cuboid | L4 |  | **Y** |
| **B:OR-14:8-893** | **FS-06-913** | Rib Fragment | L4 |  |  |
| **B:OR-14:8-894** | **FS-06-914** | Long bone fragment | L4 |  |  |
| **B:OR-14:8-895** | **FS-06-915** | Long bone fragment | L4 |  |  |
| **B:OR-14:8-896** | **FS-06-916** | Metatarsal | L4 |  |  |
| **B:OR-14:8-897** | **FS-06-917** | Rib Fragment | L4 |  |  |
| **B:OR-14:8-898** | **FS-06-918** | metacarpal | L4 |  |  |
| **B:OR-14:8-899** | **FS-06-919** | Rib Fragment | L4 |  |  |
| **B:OR-14:8-900** | **FS-06-920** | Rib Fragment | L4 |  |  |
| **B:OR-14:8-901** | **FS-06-921** | Long bone fragment | L4 |  |  |
| **B:OR-14:8-902** | **FS-06-922** | Distal Pedal phalanx | L4 |  |  |
| **B:OR-14:8-903** | **FS-06-923** | Right Lunate | L4 |  | **Y** |
| **B:OR-14:8-904** | **FS-06-924** | Long bone fragment | L4 |  |  |
| **B:OR-14:8-905** | **FS-06-925** | proximal Pedal phalanx | L4 |  |  |
| **B:OR-14:8-906** | **FS-06-926** | Non- identifiable | L4 |  |  |
| **B:OR-14:8-907** | **FS-06-927** | Vertebral Fragment | L4 |  |  |
| **B:OR-14:8-908** | **FS-06-928** | metacarpal | L4 |  |  |
| **B:OR-14:8-909** | **FS-06-929** | Long bone fragment | L4 |  |  |
| **B:OR-14:8-910** | **FS-06-930** | Rib Fragment | L4 |  |  |
| **B:OR-14:8-911** | **FS-06-931** | Long bone fragment | L4 |  |  |
| **B:OR-14:8-912** | **FS-06-932** | Rib Fragment | L4 |  |  |
| **B:OR-14:8-913** | **FS-06-933** | Cranial vault fragment | L4 |  |  |
| **B:OR-14:8-914** | **FS-06-934** | Cranial vault fragment | L4 |  |  |
| **B:OR-14:8-915** | **FS-06-935** | Long bone fragment | L4 |  |  |
| **B:OR-14:8-916** | **FS-06-936** | Long bone fragment | L4 |  |  |
| **B:OR-14:8-917** | **FS-06-937** | Vertebral Fragment | L4 |  |  |
| **B:OR-14:8-918** | **FS-06-938** | Long bone fragment | L4 |  |  |
| **B:OR-14:8-919** | **FS-06-939** | Hand phalanx | L4 |  |  |
| **B:OR-14:8-920** | **FS-06-940** | Rib Fragment | L4 |  |  |
| **B:OR-14:8-921** | **FS-06-941** | Vertebral Fragment | L4 |  |  |
| **B:OR-14:8-922** | **FS-06-942** | Non- identifiable | L4 |  |  |
| **B:OR-14:8-923** | **FS-06-943** | Interm. Hand phalanx | L4 |  |  |
| **B:OR-14:8-924** | **FS-06-944** | Rib Fragment | L4 |  |  |
| **B:OR-14:8-925** | **FS-06-945** | Long bone fragment | L4 |  |  |
| **B:OR-14:8-926** | **FS-06-946** | Long bone fragment | L4 |  |  |
| **B:OR-14:8-927** | **FS-06-947** | Long bone fragment | L4 |  |  |
| **B:OR-14:8-928** | **FS-06-948** | Distal hand phalanx | L4 |  |  |
| **B:OR-14:8-929** | **FS-06-949** | Long bone fragment | L4 |  |  |
| **B:OR-14:8-930** | **FS-06-950** | Vertebral Fragment | L4 |  |  |
| **B:OR-14:8-931** | **FS-06-951** | Rib Fragment | L4 |  |  |
| **B:OR-14:8-932** | **FS-06-952** | Rib Fragment | L4 |  |  |
| **B:OR-14:8-933** | **FS-06-953** | Temporal fragment | L4 |  |  |
| **B:OR-14:8-934** | **FS-06-954** | Hand phalanx | L4 |  |  |
| **B:OR-14:8-935** | **FS-06-955** | Non- identifiable | L4 |  |  |
| **B:OR-14:8-936** | **FS-06-956** | Long bone fragment | L4 |  |  |
| **B:OR-14:8-937** | **FS-06-957** | Rib Fragment | L4 |  |  |
| **B:OR-14:8-938** | **FS-06-958** | Long bone fragment | L4 |  |  |
| **B:OR-14:8-939** | **FS-06-959** | Rib Fragment | L4 |  |  |
| **B:OR-14:8-940** | **FS-06-960** | Non- identifiable | L4 |  |  |
| **B:OR-14:8-941** | **FS-06-961** | Cranial vault fragment | L4 |  |  |
| **B:OR-14:8-942** | **FS-06-962** | Long bone fragment | L4 |  |  |
| **B:OR-14:8-943** | **FS-06-963** | Long bone fragment | L4 |  |  |
| **B:OR-14:8-944** | **FS-06-964** | Non- identifiable | L4 |  |  |
| **B:OR-14:8-945** | **FS-06-965** | Left Scaphoid process | L4 |  |  |
| **B:OR-14:8-946** | **FS-06-966** | Rib Fragment | L4 |  |  |
| **B:OR-14:8-947** | **FS-06-967** | Rib Fragment | L4 |  |  |
| **B:OR-14:8-948** | **FS-06-968** | Long bone fragment | L4 |  |  |
| **B:OR-14:8-949** | **FS-06-969** | Rib Fragment | L4 |  |  |
| **B:OR-14:8-950** | **FS-06-970** | Rib Fragment | L4 |  |  |
| **B:OR-14:8-951** | **FS-06-971** | Cranial vault fragment | L4 |  |  |
| **B:OR-14:8-952** | **FS-06-972** | Non- identifiable | L4 |  |  |
| **B:OR-14:8-953** | **FS-06-973** | Non- identifiable | L4 |  |  |
| **B:OR-14:8-954** | **FS-06-974** | Long bone fragment | L4 |  |  |
| **B:OR-14:8-955** | **FS-06-975** | Long bone fragment | L4 |  |  |
| **B:OR-14:8-956** | **FS-06-976** | Rib Fragment | L4 |  |  |
| **B:OR-14:8-957** | **FS-06-977** | Cranial vault fragment | L4 |  |  |
| **B:OR-14:8-958** | **FS-06-978** | Long bone fragment | L4 |  |  |
| **B:OR-14:8-959** | **FS-06-979** | Rib Fragment | L4 |  |  |
| **B:OR-14:8-960** | **FS-06-980** | Non- identifiable | L4 |  |  |
| **B:OR-14:8-961** | **FS-06-981** | Non- identifiable | L4 |  |  |
| **B:OR-14:8-962** | **FS-06-982** | Vertebral Fragment | L4 |  |  |
| **B:OR-14:8-963** | **FS-06-983** | Vertebral Fragment | L4 |  |  |
| **B:OR-14:8-964** | **FS-06-984** | Non- identifiable | L4 |  |  |
| **B:OR-14:8-965** | **FS-06-985** | Long bone fragment | L4 |  |  |
| **B:OR-14:8-966** | **FS-06-986** | Long bone fragment | L4 |  |  |
| **B:OR-14:8-967** | **FS-06-987** | Long bone fragment | L4 |  |  |
| **B:OR-14:8-968** | **FS-06-988** | Non- identifiable | L4 |  |  |
| **B:OR-14:8-969** | **FS-06-989** | Non- identifiable | L4 |  |  |
| **B:OR-14:8-970** | **FS-06-990** | Cranial vault fragment | L4 |  |  |
| **B:OR-14:8-971** | **FS-06-991** | Rib Fragment | L4 |  |  |
| **B:OR-14:8-972** | **FS-06-992** | Cranial vault fragment | L4 |  |  |
| **B:OR-14:8-973** | **FS-06-993** | Rib Fragment | L4 |  |  |
| **B:OR-14:8-974** | **FS-06-994** | Cranial vault fragment | L4 |  |  |
| **B:OR-14:8-975** | **FS-06-995** | Rib Fragment | L4 |  |  |
| **B:OR-14:8-976** | **FS-06-996** | Rib Fragment | L4 |  |  |
| **B:OR-14:8-977** | **FS-06-997** | Rib Fragment | L4 |  |  |
| **B:OR-14:8-978** | **FS-06-998** | Long bone fragment | L4 |  |  |
| **B:OR-14:8-979** | **FS-06-999** | Non- identifiable | L4 |  |  |
| **B:OR-14:8-980** | **FS-06-1000** | Rib Fragment | L4 |  |  |
| **B:OR-14:8-981** | **FS-06-1001** | Non- identifiable | L4 |  |  |
| **B:OR-14:8-982** | **FS-06-1002** | Cranial vault fragment | L4 |  |  |
| **B:OR-14:8-983** | **FS-06-1003** | Rib Fragment | L4 |  |  |
| **B:OR-14:8-984** | **FS-06-1004** | Non- identifiable | L4 |  |  |
| **B:OR-14:8-985** | **FS-06-1005** | Non- identifiable | L4 |  |  |
| **B:OR-14:8-986** | **FS-06-1006** | Long bone fragment | L4 |  |  |
| **B:OR-14:8-987** | **FS-06-1007** | Long bone fragment | L4 |  |  |
| **B:OR-14:8-988** | **FS-06-1008** | Long bone fragment | L4 |  |  |
| **B:OR-14:8-989** | **FS-06-1009** | Non- identifiable | L4 |  |  |
| **B:OR-14:8-990** | **FS-06-1010** | Non- identifiable | L4 |  |  |
| **B:OR-14:8-991** | **FS-06-1011** | Rib Fragment | L4 |  |  |
| **B:OR-14:8-992** | **FS-06-1012** | Rib Fragment | L4 |  |  |
| **B:OR-14:8-993** | **FS-06-1013** | Rib Fragment | L4 |  |  |
| **B:OR-14:8-994** | **FS-06-1014** | Rib Fragment | L4 |  |  |
| **B:OR-14:8-995** | **FS-06-1015** | Non- identifiable | L4 |  |  |
| **B:OR-14:8-996** | **FS-06-1016** | Rib Fragment | L4 |  |  |
| **B:OR-14:8-997** | **FS-06-1017** | Long bone fragment | L4 |  |  |
| **B:OR-14:8-998** | **FS-06-1018** | Non- identifiable | L4 |  |  |
| **B:OR-14:8-999** | **FS-06-1019** | Non- identifiable | L4 |  |  |
| **B:OR-14:8-1000** | **FS-06-1020** | Rib Fragment | L4 |  |  |
| **B:OR-14:8-1001** | **FS-06-1021** | Non- identifiable | L4 |  |  |
| **B:OR-14:8-1002** | **FS-06-1022** | Non- identifiable | L4 |  |  |
| **B:OR-14:8-1003** | **FS-06-1023** | Non- identifiable | L4 |  |  |
| **B:OR-14:8-1004** | **FS-06-1024** | Non- identifiable | L4 |  |  |
| **B:OR-14:8-1005** | **FS-06-1025** | LI^2^ | L4 |  | **Y** |
| **B:OR-14:8-1006** | **FS-06-1026** | LI^1^ | L4 |  | **Y** |
| **B:OR-14:8-1007** | **FS-06-1027** | RM_1_ | L4 |  | **Y** |
| **B:OR-14:8-1008** | **FS-06-1028** | RM^2^ | L4 |  | **Y** |
| **B:OR-14:8-1009** | **FS-06-1029** | Premolar root | L4 |  |  |
| **B:OR-14:8-1010** | **FS-06-1030** | LM_1_ | L5 | 93-103cm | **Y** |
| **B:OR-14:8-1011** | **FS-06-1031** | RI_2_ | L5 |  | **Y** |
| **B:OR-14:8-1012** | **FS-06-1032** | Long bone fragment | L5 |  |  |
| **B:OR-14:8-1013** | **FS-06-1033** | Non- identifiable | L5 |  |  |
| **B:OR-14:8-1014** | **FS-06-1034** | Long bone fragment | L5 |  |  |
| **B:OR-14:8-1015** | **FS-06-1035** | Phalanx | L5 |  |  |
| **B:OR-14:8-1016** | **FS-06-1036** | Maxillary fragment | L4 |  | **Y** |
| **B:OR-14:8-1017** | **FS-06-1037** | Long bone fragment | L5 |  |  |
| **B:OR-14:8-1018** | **FS-06-1038** | Carpal | L5 |  |  |
| **B:OR-14:8-1019** | **FS-06-1039** | Long bone fragment | L5 |  |  |
| **B:OR-14:8-1020** | **FS-06-1040** | Bone Fragment | L5 |  |  |
| **B:OR-14:8-1021** | **FS-06-1041** | Long bone fragment | L5 |  |  |
| **B:OR-14:8-1022** | **FS-06-1042** | Long bone fragment | L5 |  |  |
| **B:OR-14:8-1023** | **FS-06-1043** | Long bone fragment | L5 |  |  |
| **B:OR-14:8-1024** | **FS-06-1044** | Non- identifiable | L5 |  |  |
| **B:OR-14:8-1025** | **FS-06-1045** | Long bone fragment | L5 |  |  |
| **B:OR-14:8-1026** | **FS-06-1046** | Cranial vault fragment | L5 |  |  |
| **B:OR-14:8-1027** | **FS-06-1047** | Hand phalanx | L5 |  |  |
| **B:OR-14:8-1028** | **FS-06-1048** | Phalanx | L5 |  |  |
| **B:OR-14:8-1029** | **FS-06-1049** | Non- identifiable | L5 |  |  |
| **B:OR-14:8-1030** | **FS-06-1050** | Long bone fragment | L5 |  |  |
| **B:OR-14:8-1031** | **FS-06-1051** | Phalanx | L5 |  |  |
| **B:OR-14:8-1032** | **FS-06-1052** | Non- identifiable | L5 |  |  |
| **B:OR-14:8-1033** | **FS-06-1053** | Phalanx | L5 |  |  |
| **B:OR-14:8-1034** | **FS-06-1054** | Pedal Phalanx | L5 |  |  |
| **B:OR-14:8-1035** | **FS-06-1055** | Right Trapezoid | L5 |  | **Y** |
| **B:OR-14:8-1036** | **FS-06-1056** | Phalanx | L5 |  |  |
| **B:OR-14:8-1037** | **FS-06-1057** | Long bone fragment | L5 |  |  |
| **B:OR-14:8-1038** | **FS-06-1058** | Long bone fragment | L5 |  |  |
| **B:OR-14:8-1039** | **FS-06-1059** | Cranial vault fragment | L5 |  |  |
| **B:OR-14:8-1040** | **FS-06-1060** | Non- identifiable | L5 |  |  |
| **B:OR-14:8-1041** | **FS-06-1061** | Long bone fragment | L5 |  |  |
| **B:OR-14:8-1042** | **FS-06-1062** | Long bone fragment | L5 |  |  |
| **B:OR-14:8-1043** | **FS-06-1063** | Non- identifiable | L5 |  |  |
| **B:OR-14:8-1044** | **FS-06-1064** | Long bone fragment | L5 |  |  |
| **B:OR-14:8-1045** | **FS-06-1065** | Long bone fragment | L5 |  |  |
| **B:OR-14:8-1046** | **FS-06-1066** | Non- identifiable | L5 |  |  |
| **B:OR-14:8-1047** | **FS-06-1067** | Long bone fragment | L5 |  |  |
| **B:OR-14:8-1048** | **FS-06-1068** | Long bone fragment | L5 |  |  |
| **B:OR-14:8-1049** | **FS-06-1069** | Long bone fragment | L5 |  |  |
| **B:OR-14:8-1050** | **FS-06-1070** | Rib Fragment | L5 |  |  |
| **B:OR-14:8-1051** | **FS-06-1071** | Long bone fragment | L5 |  |  |
| **B:OR-14:8-1052** | **FS-06-1072** | Cranial vault fragment | L5 |  |  |
| **B:OR-14:8-1053** | **FS-06-1073** | Rib Fragment | L5 |  |  |
| **B:OR-14:8-1054** | **FS-06-1074** | Non- identifiable | L5 |  |  |
| **B:OR-14:8-1055** | **FS-06-1075** | Long bone fragment | L5 |  |  |
| **B:OR-14:8-1056** | **FS-06-1076** | Long bone fragment | L5 |  |  |
| **B:OR-14:8-1057** | **FS-06-1077** | Long bone fragment | L5 |  |  |
| **B:OR-14:8-1058** | **FS-06-1078** | Long bone fragment | L5 |  |  |
| **B:OR-14:8-1059** | **FS-06-1079** | Long bone fragment | L5 |  |  |
| **B:OR-14:8-1060** | **FS-06-1080** | Long bone fragment | L5 |  |  |
| **B:OR-14:8-1061** | **FS-06-1081** | Long bone fragment | L5 |  |  |
| **B:OR-14:8-1062** | **FS-06-1082** | Long bone fragment | L5 |  |  |
| **B:OR-14:8-1063** | **FS-06-1083** | Long bone fragment | L5 |  |  |
| **B:OR-14:8-1064** | **FS-06-1084** | Long bone fragment | L5 |  |  |
| **B:OR-14:8-1065** | **FS-06-1085** | Long bone fragment | L5 |  |  |
| **B:OR-14:8-1066** | **FS-06-1086** | Non- identifiable | L5 |  |  |
| **B:OR-14:8-1067** | **FS-06-1087** | Non- identifiable | L5 |  |  |
| **B:OR-14:8-1068** | **FS-06-1088** | Non- identifiable | L5 |  |  |
| **B:OR-14:8-1069** | **FS-06-1089** | Non- identifiable | L5 |  |  |
| **B:OR-14:8-1070** | **FS-06-1090** | Non- identifiable | L5 |  |  |
| **B:OR-14:8-1071** | **FS-06-1091** | Non- identifiable | L5 |  |  |
| **B:OR-14:8-1072** | **FS-06-1092** | Non- identifiable | L5 |  |  |
| **B:OR-14:8-1073** | **FS-06-1093** | Non- identifiable | L5 |  |  |
| **B:OR-14:8-1074** | **FS-06-1094** | Non- identifiable | L5 |  |  |
| **B:OR-14:8-1075** | **FS-06-1095** | Non- identifiable | L5 |  |  |
| **B:OR-14:8-1076** | **FS-06-1096** | Non- identifiable | L5 |  |  |
| **B:OR-14:8-1077** | **FS-06-1097** | Non- identifiable | L5 |  |  |
| **B:OR-14:8-1078** | **FS-06-1098** | Non- identifiable | L5 |  |  |
| **B:OR-14:8-1079** | **FS-06-1099** | Non- identifiable | L5 |  |  |
| **B:OR-14:8-1080** | **FS-06-1100** | Cranial vault fragment | L5 |  |  |
| **B:OR-14:8-1081** | **FS-06-1101** | Right humerus |  |  | **Y** |
