# Supplementary material for: Small-Bodied Humans from Palau, Micronesia
Source: PLoS One. 2008 Mar 12;3(3):e1780. doi: 10.1371/journal.pone.0001780 (PMC2268239; doi:10.1371/journal.pone.0001780)
Supplement: Supplementary Data S3 — A summary table of the NISP (number of individual specimens) collected from Omedokel Cave as well as a complete list of all specimens found in Omedokel cave indicating whether the specimens were measurable as well as those specimens used for AMS and DNA analysis. (0.27 MB DOC [file pone.0001780.s003.doc]

Supplementary Data 3: A summary table of the NISP (number of individual specimens) collected from Omedokel Cave as well as a complete list of all specimens found in Omedokel cave indicating whether the specimens were measurable as well as those specimens used for AMS and DNA analysis.

| **Element(s)** | **Surface** |
| --- | --- |
| **Cranium** | 27 |
| **Mandible** | 7 |
| **Vertebra** | 1 |
| **Rib** | 0 |
| **Sternum** | 0 |
| **Scapula** | 0 |
| **Clavicle** | 0 |
| **Humerus** | 6 |
| **Radius** | 3 |
| **Ulna** | 4 |
| **Carpals** | 0 |
| **Hand phalanges** | 0 |
| **Os coxa** | 4 |
| **Sacrum/coccyx** | 0 |
| **Femur** | 3 |
| **Patella** | 0 |
| **Tibia** | 3 |
| **Fibula** | 4 |
| **Tarsals** | 11 |
| **Foot phalanges** | 1 |
| **Dentition** | 36 |
| **Other** | 0 |
| ***SUBTOTAL*** | **110** |
| **Number measurable specimens** | **87** |
| **Unidentifiable fragments** | 0 |
| ***TOTAL*** | **110** |

| **Omedokel Cave** |  |  |  |  |
| --- | --- | --- | --- | --- |
| **Museum Specimen number** | **Field allocation number** | **Description** | **Comments** | **Measurements taken?** |
| **B:OR-15:18-001** | **OM-001** | Left Mandible |  | **Y** |
| **B:OR-15:18-002** | **OM-001B** | RC_1_ |  | **Y** |
| **B:OR-15:18-003** | **OM-001C** | RP_3_ |  | **Y** |
| **B:OR-15:18-004** | **OM-002** | Right Talus |  | **Y** |
| **B:OR-15:18-005** | **OM-003** | Large frontal |  | **Y** |
| **B:OR-15:18-006** | **OM-004** | Right Mandibular fragment | Has ramus and impacted M_3_ | **Y** |
| **B:OR-15:18-007** | **OM-005** | Left Mandible | 2 teeth in situ M_3_ is impacted | **Y** |
| **B:OR-15:18-008** | **OM-006** | Right Mandible |  | **Y** |
| **B:OR-15:18-009** | **OM-007** | Female pelvis | Some calcrete over acetabulum | **Y** |
| **B:OR-15:18-010** | **OM-008** | Left Talus |  | **Y** |
| **B:OR-15:18-011** | **OM-009** | Left Talus |  | **Y** |
| **B:OR-15:18-012** | **OM-010** | Fragmented Mandible |  | **Y** |
| **B:OR-15:18-013** | **OM-011a** | R Femoral Head | Fragment | **Y** |
| **B:OR-15:18-014** | **OM-011b** | Right Distal Humerus |  | **Y** |
| **B:OR-15:18-015** | **OM-011c** | Left Distal Humerus |  | **Y** |
| **B:OR-15:18-016** | **OM-012** | Left Radius fragment |  | **Y** |
| **B:OR-15:18-017** | **OM-013** | RI^2^ |  | **Y** |
| **B:OR-15:18-018** | **OM-014a** | RM_2_ |  | **Y** |
| **B:OR-15:18-019** | **OM-014b** | LP_3_ |  | **Y** |
| **B:OR-15:18-020** | **OM-015** | Right Distal fibula |  | **Y** |
| **B:OR-15:18-021** | **OM-016** | Left Distal Tibia |  | **Y** |
| **B:OR-15:18-022** | **OM-017a** | LP_3_ |  | **Y** |
| **B:OR-15:18-023** | **OM-017b** | LM_2_ |  | **Y** |
| **B:OR-15:18-024** | **OM-018** | Left Distal Humerus |  | **Y** |
| **B:OR-15:18-025** | **OM-019A** | LC_1_ |  | **Y** |
| **B:OR-15:18-026** | **OM-019B** | RM_1_ |  | **Y** |
| **B:OR-15:18-027** | **OM-020** | Right Distal ulna |  | **Y** |
| **B:OR-15:18-028** | **OM-021** | Right Distal fibula |  | **Y** |
| **B:OR-15:18-029** | **OM-022** | Right Calcaneus |  | **Y** |
| **B:OR-15:18-030** | **OM-023** | RP_3_ |  | **Y** |
| **B:OR-15:18-031** | **OM-024** | Right Proximal Ulna |  | **Y** |
| **B:OR-15:18-032** | **OM-025** | RP^3^ |  | **Y** |
| **B:OR-15:18-034** | **OM-027a** | Right Calcaneus |  | **Y** |
| **B:OR-15:18-035** | **OM-027b** | Right Distal Lateral condyl of femur |  | **Y** |
| **B:OR-15:18-036** | **OM-027g** | Mandible | Right side fragm with M_1_ and sockets C_1_ - M_2_ | **Y** |
| **B:OR-15:18-037** | **OM-028** | Right Talus |  | **Y** |
| **B:OR-15:18-038** | **OM-029a** | Right Talus |  | **Y** |
| **B:OR-15:18-039** | **OM-029b** | Right Talus |  | **Y** |
| **B:OR-15:18-040** | **OM-029c** | Left Proximal Tibia |  | **Y** |
| **B:OR-15:18-041** | **OM-029d** | Right Calcaneus |  | **Y** |
| **B:OR-15:18-042** | **OM-029e** | Left Calcaneus |  | **Y** |
| **B:OR-15:18-043** | **OM-029f** | Left Distal fibula |  | **Y** |
| **B:OR-15:18-044** | **OM-029g** | Pelvis Fragment |  | **Y** |
| **B:OR-15:18-045** | **OM-030a** | Right Distal Tibia |  | **Y** |
| **B:OR-15:18-046** | **OM-030b** | Right Proximal Humerus |  | **Y** |
| **B:OR-15:18-047** | **OM-031a** | Left Distal ulna |  | **Y** |
| **B:OR-15:18-048** | **OM-031b** | Right Distal fibula |  | **Y** |
| **B:OR-15:18-049** | **OM-031c** | Right Proximal Ulna |  | **Y** |
| **B:OR-15:18-050** | **OM-031d** | Right Proximal Radius |  | **Y** |
| **B:OR-15:18-051** | **OM-032** | Maxilla | Left with I^2^ -P^4^ (and sockets of M^1/2^) | **Y** |
| **B:OR-15:18-052a** | **OM-033A** | RC^1^ |  | **Y** |
| **B:OR-15:18-052b** | **OM-033B** | RI^2^ | Very worn | **Y** |
| **B:OR-15:18-053** | **OM-034** | LM^1^ |  | **Y** |
| **B:OR-15:18-054** | **OM-034a** | Left Distal Humerus |  | **Y** |
| **B:OR-15:18-055a** | **OM-035A** | Maxilla | Right fragm with P^3^-M^1^ | **Y** |
| **B:OR-15:18-055b** | **OM-035B** | Maxilla | Left fragm with C^1^-M^1^ assoc with OM-035a | **Y** |
| **B:OR-15:18-056** | **OM- 045** | Lateral Metatarsal | (om-31I) |  |
| **B:OR-15:18-057** | **OM- 046** | Ischial tuberosity fragment | Heavily weathered |  |
| **B:OR-15:18-058** | **OM- 047** | Left Calcaneus | Heavily weathered |  |
| **B:OR-15:18-059** | **OM-100** | RI_1_ |  | **Y** |
| **B:OR-15:18-060** | **OM-101** | LI_2_ |  | **Y** |
| **B:OR-15:18-061** | **OM-102** | LC_1_ |  | **Y** |
| **B:OR-15:18-062** | **OM-103** | LP_4_ |  | **Y** |
| **B:OR-15:18-063** | **OM-104** | LM_2_ |  | **Y** |
| **B:OR-15:18-064** | **OM-105** | RM_1_ |  | **Y** |
| **B:OR-15:18-065** | **OM-106** | RM^2^ |  | **Y** |
| **B:OR-15:18-066** | **OM-107** | M_1_ |  | **Y** |
| **B:OR-15:18-067** | **OM-108** | LI^1^ |  | **Y** |
| **B:OR-15:18-068** | **OM-109** | LI^2^ |  | **Y** |
| **B:OR-15:18-069** | **OM-110** | LC^1^ |  | **Y** |
| **B:OR-15:18-070** | **OM-111** | RC^1^ |  | **Y** |
| **B:OR-15:18-071** | **OM-112** | RP_3_ |  | **Y** |
| **B:OR-15:18-072** | **OM-113** | RP^4^ |  | **Y** |
| **B:OR-15:18-073** | **OM-114** | RM^2^ |  | **Y** |
| **B:OR-15:18-074** | **OM-115** | RM^2^ |  | **Y** |
| **B:OR-15:18-075** | **OM-116** | LM^1^ |  | **Y** |
| **B:OR-15:18-076** | **OM-117** | RM^1^ |  | **Y** |
| **B:OR-15:18-077** | **OM-118** | RM^2^ |  | **Y** |
| **B:OR-15:18-078** | **OM-119** | LM^3^ |  | **Y** |
| **B:OR-15:18-079** | **OM-120** | LM |  | **Y** |
| **B:OR-15:18-080** | **OM-501** | Frontal fragment |  | **Y** |
| **B:OR-15:18-081** | **OM-502** | Frontal fragment |  | **Y** |
| **B:OR-15:18-082** | **OM-503** | Left Maxilla | Teeth present: LC^1^,LP^3/4^ | **Y** |
| **B:OR-15:18-083** | **OM-504** | Complete mandible | Teeth skew with staining RI_1/2_, RM_1/2_ -LC LP_3_ LP_4_ LM_1/2_ Measurements taken on all teeth in mandible | **Y** |
| **B:OR-15:18-084** | **OM-505** | Maxilla | Contains teeth: RI^2^,RC^1^ RP^3/4^, RM^1^ on LC^1^, P^3/4^, LM^1/2^ | **Y** |
| **B:OR-15:18-086** | **OM-507** | Left maxillary fragment | has LP^4^ and sockets for LI^1^-LP^3^ | **Y** |
| **B:OR-15:18-087** | **OM-508** | Pelvis | Male narrow siatic notch oval foramen. pubic body square.no ventral arc | **Y** |
| **B:OR-15:18-088** | **OM-509** | Left Distal Humerus fragment |  | **Y** |
| **B:OR-15:18-089** | **OM-029H** | Left Femoral head fragment |  | **Y** |
| **B:OR-15:18-090** | **OM-29G2** | Distal Radius |  | **Y** |
| **B:OR-15:18-091** | **OM-600** | Large cranial vault fragment | Collected from Omdokel cave by M.Etpison |  |
| **B:OR-15:18-092** | **OM-601** | Large cranial vault fragment | Collected from Omdokel cave by M.Etpison |  |
| **B:OR-15:18-093** | **OM-602** | Large Basal cranial fragment | Collected from Omdokel cave by M.Etpison |  |
| **B:OR-15:18-094** | **OM-603** | Large Basal cranial fragment | Collected from Omdokel cave by M.Etpison |  |
| **B:OR-15:18-095** | **OM-604** | Cranial fragment | Collected from Omdokel cave by M.Etpison |  |
| **B:OR-15:18-096** | **OM-605** | Cranial fragment | Collected from Omdokel cave by M.Etpison |  |
| **B:OR-15:18-097** | **OM-606** | Cranial fragment | Collected from Omdokel cave by M.Etpison |  |
| **B:OR-15:18-098** | **OM-607** | Cranial fragment | Collected from Omdokel cave by M.Etpison |  |
| **B:OR-15:18-099** | **OM-608** | Cranial fragment | Collected from Omdokel cave by M.Etpison |  |
| **B:OR-15:18-100** | **OM-609** | Cranial fragment | Collected from Omdokel cave by M.Etpison |  |
| **B:OR-15:18-101** | **OM-610** | Cranial fragment | Collected from Omdokel cave by M.Etpison |  |
| **B:OR-15:18-102** | **OM-611** | Cranial fragment | Collected from Omdokel cave by M.Etpison |  |
| **B:OR-15:18-103** | **OM-612** | Cranial fragment | Collected from Omdokel cave by M.Etpison |  |
| **B:OR-15:18-104** | **OM-613** | Cranial fragment | Collected from Omdokel cave by M.Etpison |  |
| **B:OR-15:18-105** | **OM-614** | Cranial fragment | Collected from Omdokel cave by M.Etpison |  |
| **B:OR-15:18-106** | **OM-615** | Cranial fragment | Collected from Omdokel cave by M.Etpison |  |
| **B:OR-15:18-107** | **OM-616** | Vertebra | Collected from Omdokel cave by M.Etpison |  |
| **B:OR-15:18-108** | **OM-617** | Maxillary fragment | Collected from Omdokel cave by M.Etpison |  |
| **B:OR-15:18-109** | **OM-618** | Maxillary fragment with Molar | Collected from Omdokel cave by M.Etpison |  |
| **B:OR-15:18-110** | **OM-619** | Incisor | Collected from Omdokel cave by M.Etpison |  |
