# Supplementary material for: Small-Bodied Humans from Palau, Micronesia
Source: PLoS One. 2008 Mar 12;3(3):e1780. doi: 10.1371/journal.pone.0001780 (PMC2268239; doi:10.1371/journal.pone.0001780)
Supplement: Supplementary Data S4 — (0.19 MB DOC) [file pone.0001780.s004.doc]

**Supplementary Data 4**

| Ratios of craniofacial to postcranial dimensions in Palauans, small-bodied Africans (San), and Flores LB1^a^ | |
| --- | --- |
| Orbital breadth/Humeral distal articular breadth | |
| Palauans | 0.942 – 0.839 |
| San (Mean, SD, n) | 0.898 ± 0.063 (9) |
| San (range) | 0.823 – 1.019 |
| San (smallest/smallest – largest/largest)^b^ | *0.838 – 0.878* |
|  |  |
| Orbital breadth/Maximum acetabular diameter | |
| Palauans | 0.785 – 0.8026 |
| San (Mean, SD, n) | 0.783 ± 0.076 (9) |
| San (range) | 0.695 – 0.928 |
| San (smallest/smallest – largest/largest) | *0.754 – 0.734* |
| Flores (LB1) | 0.889 |
|  |  |
| Orbital breadth/Femoral head AP diameter | |
| Palauans | 0.859 – 0.954 |
| San (Mean, SD, n) | 0.905 ± 0.099 (10) |
| San (range) | 0.795 – 1.090 |
| San (smallest/smallest – largest/largest) | *0.880 – 0.848* |
| Flores (LB1) | 1.0159 |
|  |  |
| Orbital breadth/Tibial proximal ML diameter | |
| Palauans | 0.584 – 0.586 |
| San (Mean, SD, n) | 0.565 ± 0.061 (10) |
| San (range) | 0.483 – 0.691 |
| San (smallest/smallest – largest/largest) | *0.558 – 0.515* |
| Flores (LB1) | 0.621 |
|  |  |
| Orbital breadth/Talar length | |
| Palauans | 0.709 – 0.707 |
| San (Mean, SD, n) | 0.721 ± 0.087 (9) |
| San (range) | 0.601 – 0.873 |
| San (smallest/smallest – largest/largest) | *0.705 – 0.641* |
|  |  |
| Interorbital breadth/Humeral distal articular breadth | |
| Palauans | 0.723 – 0.658 |
| San (Mean, SD, n) | 0.589 ± 0.059 (9) |
| San (range) | 0.507 – 0.679 |
| San (smallest/smallest – largest/largest) | *0.562 – 0.635* |
|  |  |
| Interorbital breadth/Maximum acetabular diameter | |
| Palauans | 0.603 – 0.629 |
| San (Mean, SD, n) | 0.518 ± 0.049 (9) |
| San (range) | 0.451 – 0.590 |
| San (smallest/smallest – largest/largest) | *0.506 – 0.531* |
|  |  |
| Interorbital breadth/Femoral head AP diameter | |
| Palauans | 0.659 – 0.747 |
| San (Mean, SD, n) | 0.588 ± 0.058 (10) |
| San (range) | 0.518 – 0.668 |
| San (smallest/smallest – largest/largest) | *0.590 – 0.614* |
|  |  |
| Interorbital breadth/Tibial proximal ML diameter | |
| Palauans | 0.448 – 0.460 |
| San (Mean, SD, n) | 0.367 ± 0.037 (10) |
| San (range) | 0.313 – 0.424 |
| San (smallest/smallest – largest/largest) | *0.374 – 0.373* |
|  |  |
| Interorbital breadth/Talar length | |
| Palauans | 0.545 – 0.555 |
| San (Mean, SD, n) | 0.460 ± 0.056 (9) |
| San (range) | 0.380 – 0.535 |
| San (smallest/smallest – largest/largest) | *0.472 – 0.464* |
|  |  |
| Nasal breadth/Humeral distal articular breadth | |
| Palauans | 0.614 – 0.581 |
| San (Mean, SD, n) | 0.617 ± 0.060 (9) |
| San (range) | 0.509 - 0.716 |
| San (smallest/smallest – largest/largest) | *0.577 – 0.659* |
|  |  |
| Nasal breadth/Maximum acetabular diameter | |
| Palauans | 0.511 – 0.555 |
| San (Mean, SD, n) | 0.540 ± 0.066 (9) |
| San (range) | 0.453 – 0.659 |
| San (smallest/smallest – largest/largest) | *0.519 – 0.552* |
| Flores (LB1) | 0.583 |
|  |  |
| Nasal breadth/Femoral head AP diameter | |
| Palauans | 0.560 – 0.660 |
| San (Mean, SD, n) | 0.617 ± 0.069 (10) |
| San (range) | 0.508 – 0.708 |
| San (smallest/smallest – largest/largest) | *0.605 – 0.638* |
| Flores (LB1) | 0.667 |
|  |  |
| Nasal breadth/Tibial proximal ML diameter | |
| Palauans | 0.380 – 0.406 |
| San (Mean, SD, n) | 0.385 ± 0.041 (10) |
| San (range) | 0.318 – 0.446 |
| San (smallest/smallest – largest/largest) | *0.384 – 0.388* |
| Flores (LB1) | 0.408 |
|  |  |
| Nasal breadth/Talar length | |
| Palauans | 0.462 – 0.490 |
| San (Mean, SD, n) | 0.490 ± 0.056 (9) |
| San (range) | 0.408 – 0.563 |
| San (smallest/smallest – largest/largest) | *0.485 – 0.482* |
|  |  |
| Mandibular symphyseal height/Humeral distal articular breadth | |
| Palauans | 0.809 – 0.807 |
| San (Mean, SD, n) | 0.720 ± 0.091 (5) |
| San (range) | 0.640 – 0.874 |
| San (smallest/smallest – largest/largest) | *0.688 – 0.828* |
|  |  |
| Mandibular symphyseal height/Maximum acetabular diameter | |
| Palauans | 0.673 – 0.772 |
| San (Mean, SD, n) | 0.563 ± 0.044 (5) |
| San (range) | 0.516 – 0.630 |
| San (smallest/smallest – largest/largest) | *0.619 – 0.692* |
| Flores (LB1) | 0.778 |
|  |  |
| Mandibular symphyseal height/Femoral head AP diameter | |
| Palauans | 0.737 – 0.918 |
| San (Mean, SD, n) | 0.699 ± 0.065 (5) |
| San (range) | 0.642 – 0.803 |
| San (smallest/smallest – largest/largest) | *0.722 – 0.800* |
| Flores (LB1) | 0.889 |
|  |  |
| Mandibular symphyseal height/Tibial proximal ML diameter | |
| Palauans | 0.501 – 0.564 |
| San (Mean, SD, n) | 0.442 ± 0.042 (5) |
| San (range) | 0.410 – 0.515 |
| San (smallest/smallest – largest/largest) | *0.458 – 0.486* |
| Flores (LB1) | 0.544 |
|  |  |
| Mandibular symphyseal height/Talar height | |
| Palauans | 0.609 – 0.681 |
| San (Mean, SD, n) | 0.521 ± 0.011 (4) |
| San (range) | 0.506 – 0.532 |
| San (smallest/smallest – largest/largest) | *0.579 – 0.605* |
|  |  |
| Mandibular mental foramen height/Humeral distal articular breadth | |
| Palauans | 0.669 – 0.803 |
| San (Mean, SD, n) | 0.656 ± 0.079 (5) |
| San (range) | 0.610 – 0.795 |
| San (smallest/smallest – largest/largest) | *0.639 – 0.753* |
|  |  |
| Mandibular mental foramen height/Maximum acetabular diameter | |
| Palauans | 0.557 – 0.768 |
| San (Mean, SD, n) | 0.563 ± 0.044 (5) |
| San (range) | 0.516 – 0.629 |
| San (smallest/smallest – largest/largest) | *0.575 – 0.630* |
|  |  |
| Mandibular mental foramen height/Femoral head AP diameter | |
| Palauans | 0.609 – 0.912 |
| San (Mean, SD, n) | 0.637 ± 0.057 (5) |
| San (range) | 0.579 – 0.731 |
| San (smallest/smallest – largest/largest) | *0.670 – 0.728* |
|  |  |
| Mandibular mental foramen height/ Tibial proximal ML diameter | |
| Palauans | 0.414 – 0.561 |
| San (Mean, SD, n) | 0.403 ± 0.038 (5) |
| San (range) | 0.371 – 0.468 |
| San (smallest/smallest – largest/largest) | *0.426 – 0.442* |
|  |  |
| Mandibular mental foramen height/Talar length | |
| Palauans | 0.503 – 0.690 |
| San (Mean, SD, n) | 0.475 ± 0.023 (4) |
| San (range) | 0.456 – 0.505 |
| San (smallest/smallest – largest/largest) | *0.537 – 0.550* |
|  |  |
| Mandibular molar height/Humeral distal articular breadth | |
| Palauans | 0.666 – 0.726 |
| San (Mean, SD, n) | 0.591 ± 0.071 (5) |
| San (range) | 0.523 – 0.705 |
| San (smallest/smallest – largest/largest) | *0.569 – 0.667* |
|  |  |
| Mandibular molar height/Maximum acetabular diameter | |
| Palauans | 0.554 – 0.694 |
| San (Mean, SD, n) | 0.508 ± 0.038 (5) |
| San (range) | 0.477 – 0.558 |
| San (smallest/smallest – largest/largest) | *0.512 – 0.558* |
| Flores (LB1) | 0.569 |
|  |  |
| Mandibular molar height/Femoral head AP diameter | |
| Palauans | 0.607 – 0.825 |
| San (Mean, SD, n) | 0.574 ± 0.053 (5) |
| San (range) | 0.517 – 0.647 |
| San (smallest/smallest – largest/largest) | *0.597 – 0.645* |
| Flores (LB1) | 0.651 |
|  |  |
| Mandibular molar height/Tibial proximal ML diameter | |
| Palauans | 0.413 – 0.507 |
| San (Mean, SD, n) | 0.363 ± 0.037 (5) |
| San (range) | 0.314 – 0.415 |
| San (smallest/smallest – largest/largest) | *0.379 – 0.391* |
| Flores (LB1) | 0.398 |
|  |  |
| Mandibular molar height/Talar length | |
| Palauans | 0.501 – 0.624 |
| San (Mean, SD, n) | 0.431 ± 0.030 (4) |
| San (range) | 0.388 – 0.454 |
| San (smallest/smallest – largest/largest) | *0.478 – 0.487* |
|  |  |
| a. Samples and variables as defined in Table 1. In the case of the unassociated Palauan remains, ratios were created by pairing the smallest facial dimension with the smallest postcranial dimension for each variable, and by pairing the largest facial dimension with the largest postcranial dimension for each variable. For each ratio, the value on the left for the Palauans represents the ratio of smallest/smallest facial/postcranial values, while the value on the right represents the ratio of largest/largest measurements.  b. Ratios created by pairing the smallest San facial dimension with the smallest San postcranial dimension for each variable, and by pairing the largest facial dimension with the largest postcranial dimension for each variable in the data, to create ratios using a sampling procedure parallel to that used for the Palauan data set. Since an argument could be made that these are most appropriate ratios for comparison, these values have been italicized in the table. | |
