# Supplementary material for: Small-Bodied Humans from Palau, Micronesia
Source: PLoS One. 2008 Mar 12;3(3):e1780. doi: 10.1371/journal.pone.0001780 (PMC2268239; doi:10.1371/journal.pone.0001780)
Supplement: Supplementary Data S5 — (3.11 MB DOC) [file pone.0001780.s005.doc]

**Supplementary Data 5**

**
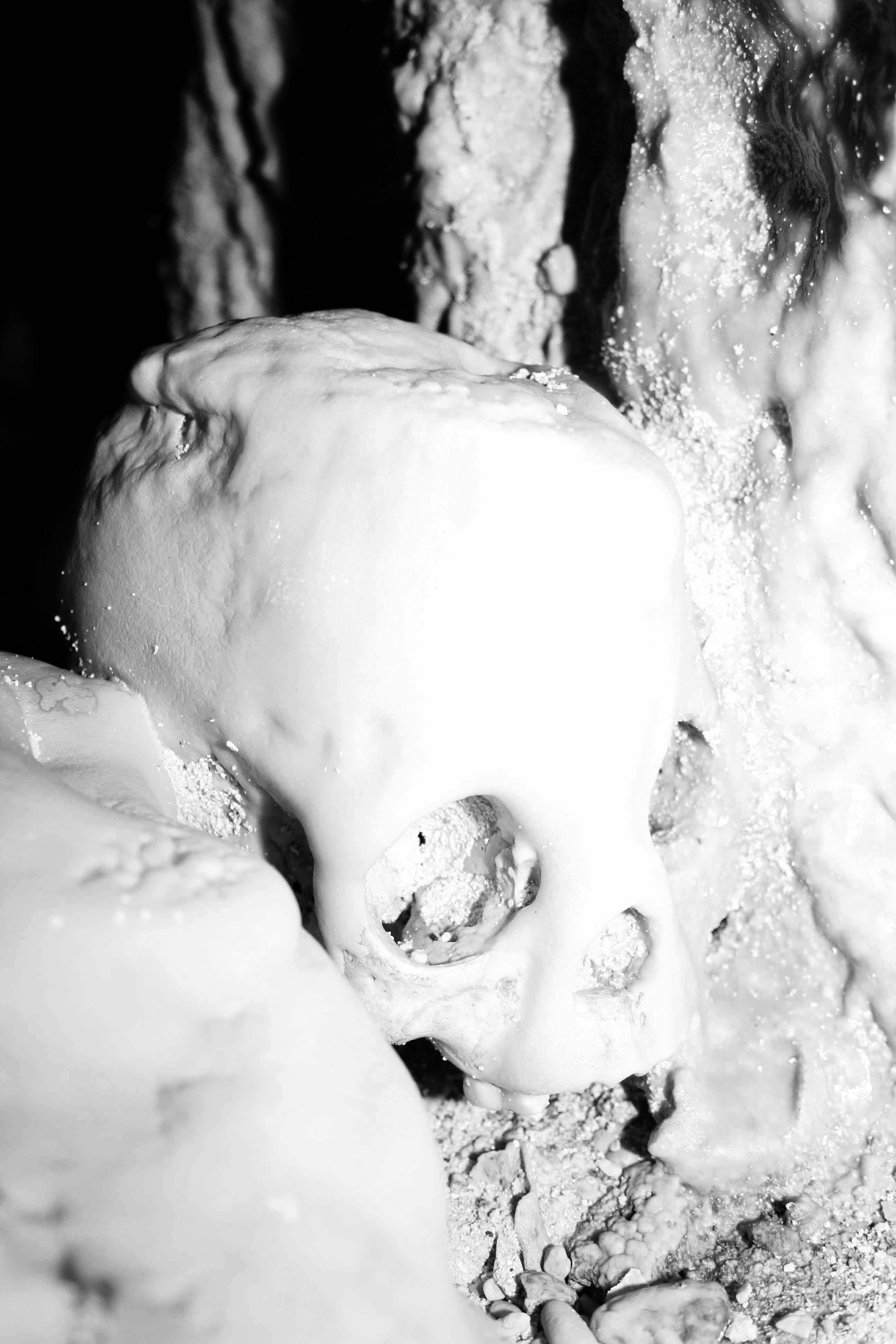
**

Figure S5 – A skull embedded in flowstone in Omedokel cave, Palau Micronesia. Although heavily encrusted with flowstone, wedged between two boulders and partially distorted, it is clear that the skull retains the same facial characters as the remainder of the sample – namely small orbits and wide glabella. Endocranial volume cannot be estimated but it is clearly small by modern small bodied human standards.
