# Supplementary material for: Small-Bodied Humans from Palau, Micronesia
Source: PLoS One. 2008 Mar 12;3(3):e1780. doi: 10.1371/journal.pone.0001780 (PMC2268239; doi:10.1371/journal.pone.0001780)
Supplement: Supplementary Data S6 — (0.05 MB DOC) [file pone.0001780.s006.doc]

**Figure S6**

**
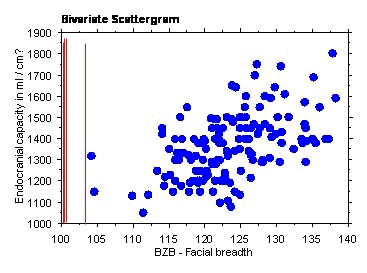
**

Correlation matrix, regression summary and Anova table on the results of 147 modern human endocranial volumes compared to facial breadth (taken across the midline of the orbits) collected from the Dart Collection, University of the Witwatersrand. The sample contains small-bodied individuals as well as large-bodied individuals from four populations (San, Zulu, Xhosa and European) males and females. Red lines indicate the estimated facial breadth of four Palauan specimens. It is highly probable that the Palauan individuals will have cranial capacities at or below the lowest range for modern humans, but we are unable to ascertain what the effect of facial size below our sample range will have on endocranial volume predictions.
