# Supplementary material for: Small-Bodied Humans from Palau, Micronesia
Source: PLoS One. 2008 Mar 12;3(3):e1780. doi: 10.1371/journal.pone.0001780 (PMC2268239; doi:10.1371/journal.pone.0001780)
Supplement: Supplementary Data S7 — (0.06 MB DOC) [file pone.0001780.s007.doc]

**Figure S7**


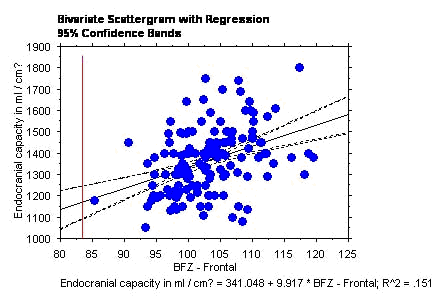


Correlation matrix, regression summary and Anova table on the results of 147 modern human endocranial volumes compared to frontal breadth. The sample comprises the same individuals measured in Supplementary Data 4. The red line indicates the estimated frontal breadth of B:OR-14: 8-001. It is highly probable that the Palauan individual will have a cranial capacity at or below the lowest range for modern humans, but we are unable to ascertain what the effect of frontal size below our sample range will have on endocranial volume predictions.
